# Past volcanic activity predisposes an endemic threatened seabird to negative anthropogenic impacts

**DOI:** 10.1038/s41598-024-52556-9

**Published:** 2024-01-23

**Authors:** Helena Teixeira, Matthieu Le Corre, Laurent Michon, Malcolm A. C. Nicoll, Audrey Jaeger, Natacha Nikolic, Patrick Pinet, François-Xavier Couzi, Laurence Humeau

**Affiliations:** 1 UMR ENTROPIE (Université de La Réunion, IRD, CNRS, IFREMER, Université de Nouvelle-Calédonie), 15 Avenue René Cassin, CS 92003, 97744 Saint Denis Cedex 9, Ile de La Réunion France; 2 Université de La Réunion, Laboratoire Géosciences Réunion, 97744 Saint Denis, France; 3 Université Paris Cité, Institut de physique du globe de Paris, CNRS, 75005 Paris, France; 4 Institute of Zoology, Zoological Society of London, Regent’s Park, London, NW1 4RY UK; 5 INRAE, AQUA, ECOBIOP, Saint-Pée-Sur-Nivelle, France; 6 Parc National de La Réunion, Life+ Pétrels, 258 Rue de la République, 97431 Plaine des Palmistes, Réunion Island France; 7 Société d’Etudes Ornithologiques de La Réunion (SEOR), 13 ruelle des Orchidées, 97440 Saint André, Réunion Island France; 8 UMR PVBMT (Université de La Réunion, CIRAD), 15 Avenue René Cassin, CS 92003, 97744 Saint Denis Cedex 9, Ile de La Réunion France

**Keywords:** Population genetics, Evolutionary biology

## Abstract

Humans are regularly cited as the main driver of current biodiversity extinction, but the impact of historic volcanic activity is often overlooked. Pre-human evidence of wildlife abundance and diversity are essential for disentangling anthropogenic impacts from natural events. Réunion Island, with its intense and well-documented volcanic activity, endemic biodiversity, long history of isolation and recent human colonization, provides an opportunity to disentangle these processes. We track past demographic changes of a critically endangered seabird, the Mascarene petrel *Pseudobulweria aterrima*, using genome-wide SNPs. Coalescent modeling suggested that a large ancestral population underwent a substantial population decline in two distinct phases, ca. 125,000 and 37,000 years ago, coinciding with periods of major eruptions of Piton des Neiges. Subsequently, the ancestral population was fragmented into the two known colonies, ca. 1500 years ago, following eruptions of Piton de la Fournaise. In the last century, both colonies declined significantly due to anthropogenic activities, and although the species was initially considered extinct, it was rediscovered in the 1970s. Our findings suggest that the current conservation status of wildlife on volcanic islands should be firstly assessed as a legacy of historic volcanic activity, and thereafter by the increasing anthropogenic impacts, which may ultimately drive species towards extinction.

## Introduction

Islands support a disproportionate amount of the world’s biodiversity, including an extraordinary level of species endemism, unique functional traits such as flightlessness in birds, and adaptive radiations e.g., Lemuriformes clade in Madagascar^1^, but they have also experienced extensive biodiversity loss^2,3^. Human impact has been identified as the primary driver of this biodiversity loss, with many islands’ endemics going extinct following human colonization and the associated habitat fragmentation and introduction of invasive species^3,4^, including the dodo *Raphus cucullatus* from Mauritius^5,6^. Although this narrative assumes that populations of island endemics were abundant before human impact, this assumption is rarely tested as little pre-human colonization information on endemic fauna abundance is available. Natural events, such as historical climatic cycles^7–10^ and island-scale volcanic eruptions^11–13^ are also known to impact species distribution and abundance worldwide^14^. Hence, disentangling anthropogenic impacts from natural events is often limited by the lack of information about historical events that may have led to population collapse before human colonization.

**Figure 1:**
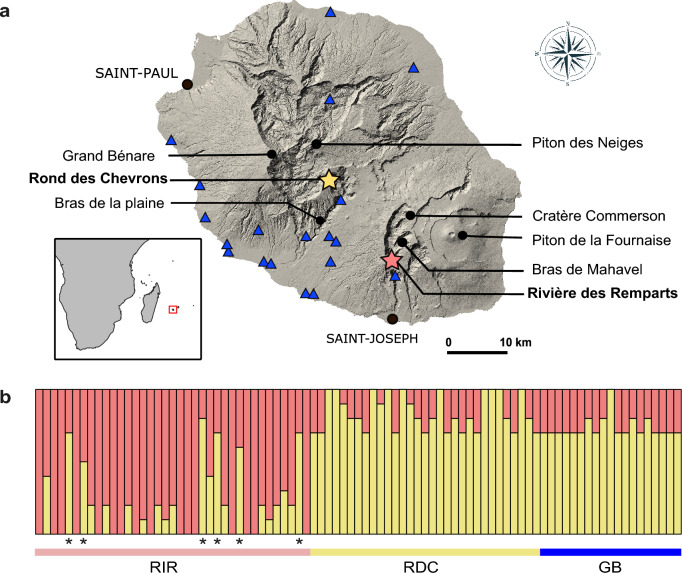
Study area and population genetic structure. (**a**) Geographic location of the two recently discovered colonies of the Mascarene petrel (*Pseudobulweria aterrima*) on Réunion Island: Rond Des Chevrons (RDC; yellow star) and Rivière des Remparts (RIR; rose star). The blue triangles represent the location of light-grounded birds (GB). (**b**) Cluster assignment of *Pseudobulweria aterrima* individuals to two genetic clusters (K = 2) using 13,855 genome-wide SNPs (n = 87 birds; dataset 1) with *Structure*. Each single vertical bar represents one individual and each color a distinct genetic cluster. Migrant birds are highlighted by an asterisk in (**b**). Sampling details can be found in Supplementary Table S1.

Réunion Island (Western Indian Ocean, WIO), with its intense and well-documented volcanic activity, endemic biodiversity, long history of isolation and comparatively recent human colonization, provides a unique opportunity to disentangle these processes. The island emerged during the late Quaternary (> 2.2 million years ago; Mya) through volcanic activity^15^, and it was likely colonized by over-sea dispersal from the neighboring islands^16^. It is composed of two volcanoes: Piton des Neiges, a dormant volcano that has been inactive for about 27 thousand years (kyr)^17^, and Piton de la Fournaise, one of the most active volcanoes on Earth (Fig. 1a). Human colonization of Réunion Island started around 400 years ago on the west coast, near what became the city of Saint-Paul^18^.

High-resolution paleoclimate records (e.g., speleothems, pollen, sediment cores, charcoal and fossils) provide unequivocal archives of pre-human species diversity and distribution, but are often limited or incomplete^19,20^. In contrast, genomes of extant organisms carry a signature of their evolutionary past, enabling the reconstruction of the demographic history of populations from the genome of their present‐day representatives^21–23^. Such long-term population data can be used to infer the potential drivers of population declines and test alternative evolutionary hypotheses^8,9,24–27^. However, even with recent developments of methods for inferring species demographic histories (reviewed in^21,28^), studies on the endemic flora and fauna of Réunion Island are limited^29–31^, and neglect the possible effects of population structure on demographic inferences^32–35^.

To address the lack of long-term population data on the endemic but critically endangered Mascarene petrel *Pseudobulweria aterrima* (family Procellariidae, Bonaparte 1857) in Réunion Island, we reconstructed its demographic history. The Mascarene petrel is one of the rarest and most threatened seabirds of the world^36^. The species was previously known from less than 10 specimens collected in Réunion Island during the nineteenth century. Evidence of its continued survival was elusive, and the species was thought to be extinct for more than eight decades, until it was rediscovered in 1970 with a dead grounded bird^37^. More recently, two breeding colonies have been discovered in Réunion Island: Rond Des Chevrons (hereafter called RDC) and Rivière des Remparts^38^ (hereafter called RIR; Fig. 1a). This species provides a good study model to investigate the impact of natural versus anthropogenic impacts in the region because (i) the Mascarene petrel has diverged from its congener Fiji Petrel (*P. macgillivrayi*) from Gau Island (Fiji) and only evolved in Réunion Island^39^; (ii) as all procellarids, the species is monogamous and highly philopatric, meaning that its population dynamics is strongly impacted by local environmental changes at breeding colonies, and by isolation between colonies^40^; (iii) the Mascarene petrel is threatened by predation by introduced mammals (rats and cats) at breeding sites and by light-induced mortality (i.e., mortality of fledglings that are attracted to artificial lights)^36,41,42^. All this suggests that the species is very susceptible to anthropogenic impacts. A genome-wide Single Nucleotide Polymorphism (SNP) dataset was generated with DArTseq for 93 birds from the two recently discovered Mascarene petrel colonies^38^ and light-grounded birds (Fig. 1a). We first investigated population structure patterns of the Mascarene petrel, and then used two complementary coalescent approaches (i.e., model-free and comparative modeling) to infer changes on effective population size (N_e_) and connectivity over time, and investigate how past demographic events were affected by natural versus anthropogenic changes. Specifically, we test whether (i) the Mascarene petrel effective population size was small and fragmented prior to substantial human impact on Réunion Island; (ii) the timing of population declines and fragmentation coincided with major volcanic or climatic events on the island; and (iii) population bottlenecks intensified during the past four decades due to increasing human impact.

## Results

### Present day population structure in Mascarene petrel

The Discriminant Analysis of Principal Components (*DAPC*), the Bayesian assignment approach implemented in *Structure,* and Wright’s F-statistics F_ST_ suggested the existence of genetic structure between the two recently discovered breeding colonies of Mascarene petrel. Both *DAPC* and *Structure* methods were congruent in inferring K = 2 as the optimal number of genetic clusters (Supplementary Figs. S1a and S2). *DAPC* clearly separated the birds from RDC and all but six birds from RIR along the first discriminant axis. All grounded birds (attracted by light pollution) were clustered together with the RDC individuals (Supplementary Fig. S1b). When using *Structure*, assuming K = 2, all the birds from RDC (n = 31) and light-grounded birds (n = 19) were assigned to one of two genetic clusters with an average membership higher than 70%. All but six birds sampled at RIR were grouped in the second cluster with an average membership ranging between 60 and 100% (Fig. 1b; see also Supplementary Fig. S3). Both methods were congruent in inferring the same six migrant birds. Additionally, substantial levels of admixture were observed in both genetic clusters, suggesting the occurrence of historic or contemporary gene flow between the two colonies (Fig. 1b). The Wright’s F-statistics F_ST_ supports a significant genetic differentiation among the two colonies, independently of the inclusion (F_ST_ = 0.0362; *p*-value = 0) or exclusion of light-grounded birds (F_ST_ = 0.0425; *p*-value = 0) in the analyses.

### Demographic history of Mascarene petrel

**Figure 2:**
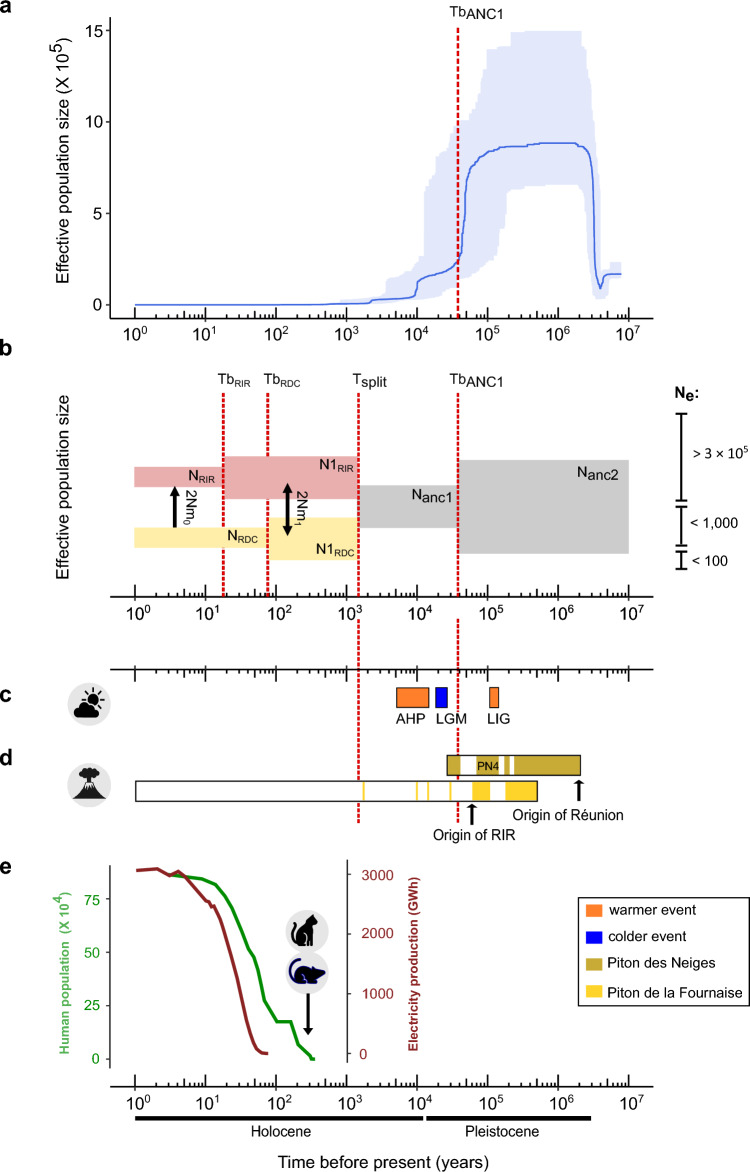
Reconstruction of the demographic history of the Mascarene petrel (*Pseudobulweria aterrima*) using two complementary coalescent approaches, and major ecological changes on Réunion Island. (**a**) Demographic history inferred with *Stairway Plot*, considering the two colonies together (n = 54 unrelated birds). The thick lines correspond to the median values of N_e_, and the ribbon represents the 95% confidence interval_._ The dashed line represents the ancient bottleneck (Tb_ANC1_) inferred by model M7 with *fastsimcoal2.* (**b**) Illustration of the most realistic model (M7; *ancient & recent bottlenecks*) revealed by *fastsimcoal2*. The occurrence of gene flow is exemplified by arrows. (**c**) Representation of the more pronounced climatic events that took place in Western Indian Ocean: LIG (Last Interglacial; ca. 132–112 kyr; kyr = thousand years), LGM (Last Glacial Maximum; ca. 26.5–19 kyr) and AHP (African Humid Period; ca. 15–5 kyr). The width of bars represents the duration of each event. (**d**) Review of the major volcanic events that took place on Réunion Island. The width of bars is proportional to the time of activity of Piton des Neiges (brown) and of the activity on the western part of Piton de la Fournaise (yellowish-orange). The arrows represent the origin of Réunion Island (> 2.2 Mya) and Rivière des Remparts valley (ca. 60 kyr). PN4 = Piton des Neiges phase 4. (**e**) Growth of the human population (green curve) and of production of electricity (dark red) at Réunion Island. Data on human population was compiled from^51,52^ and INSEE (https://www.insee.fr/fr/statistiques/2522602). Data on electricity production from Electricité de France (EDF). For (**a**) and (**b**) analyses were performed considering 2.89 × 10^−9^ as mutation rate^53^ and events were scaled considering the generation time of the Barau’s petrel (18.9 years). Population size estimates are given in number of diploid copies. RIR = Rivière des Remparts colony; RDC = Rond Des Chevrons colony; GB = light-grounded birds; N_e_ = Effective population size; N_RIR_ = N_e_ of RIR at present time; N_RDC_ = N_e_ of RDC at present time; N1_RIR_ = N_e_ of RIR after the fragmentation of the ancestral population into two colonies; N1_RDC_ = N_e_ of RDC after the fragmentation of the ancestral population into two colonies; N_ANC2_ = N_e_ of the ancestral population before size changes; T_split_ = Time of the fragmentation of the ancestral population into RIR and RDC; Tb_RIR_ = Time when RIR underwent a reduction on population size and connectivity; Tb_RDC_ = Time when RDC underwent a reduction on population size and connectivity; Tb_ANC1_ = Time when the ancestral population underwent a bottleneck; 2_Nm_ = average number of haploid immigrants entering the population per generation, where 2_Nm0_ denotes recent gene flow and 2_Nm1_ ancient gene flow among the two colonies. 2_Nm0_ = 0.01; 2_Nm1_ = 0.5.

The demographic history of the Mascarene petrel was reconstructed using two complementary coalescent approaches. The *Stairway Plot* v2.0^43^ was firstly used to infer changes on effective population size through time. Since a species' demographic history can be complicated by demographic events other than changes in population size (e.g., population splits or connectivity changes), the composite-likelihood approach implemented in *fastsimcoal2* v.2.6^44^ was additionally used to test and compare a set of demographic models incorporating population splits, resizes, and connectivity changes^9,29,45,46^. The *Stairway Plot* and *fastsimcoal2* are also known to differ on the evolutionary timescales, with the first method being more informative about hundreds of generations, and the latter about the recent past^47–50^. The two coalescent methods revealed that Mascarene petrel underwent significant demographic changes during the late Quaternary. Assuming that individuals were part of panmictic populations, the *Stairway Plot* analyses for each genetic cluster (Supplementary Fig. S4a–c) and for the entire dataset (Fig. 2a) inferred a population maxima ca. 1 Mya, that was preceded by a period of a large and rather constant N_e_ (> 8 × 10^5^ birds) until ca. 125 kyr. This event was then followed by a continuous population decline until recent times. The analyses suggest a larger N_e_ for the RDC, even when considering the same number of individuals in the analyses (n = 33 per genetic cluster; Supplementary Fig. S4c). The *Stairway Plot* runs without closely-related individuals (i.e., parent–offspring and full-sibs; n = 35 for RDC and light-grounded birds, and 19 for RIR) revealed an identical demographic history when using the complete datasets (Supplementary Fig. S4d).

**Figure 3:**
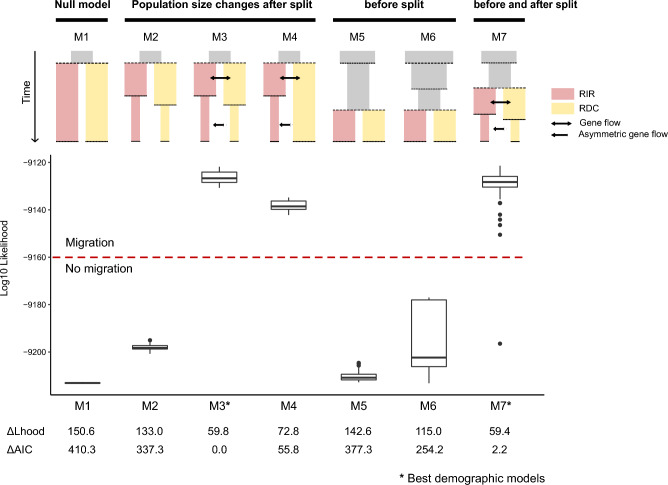
Comparison of the seven demographic models tested with *fastsimcoal2* for *Pseudobulweria aterrima.* Boxplots show the log_10_ Likelihood distributions computed based on the parameters that maximized the likelihood of each model in a total of 100 independent simulations per model. The models were ranked based on the Akaike Information Criteria (AIC). The values below the boxplots denote the ΔLhood (i.e., difference between the observed Likelihood and the maximum expected Likelihood) and ΔAIC (i.e., difference in AIC to the best demographic model) for each model. The best ranked demographic models are highlighted by an asterisk. Models M3 and M7 yielded a similar log_10_ Likelihood distribution and ΔAIC, suggesting a similar model fit. Analyses were performed using 9897 genome-wide SNPs called for 20 unrelated individuals (10 from each colony). M1 = *null model*; M2 = *bottlenecks without gene flow*; M3 = *bottlenecks with gene flow*; M4 = *asymmetric bottleneck*; M5 = *ancient bottleneck*; M6 = *two ancient bottlenecks*; M7 = *ancient and recent bottlenecks*; RIR = Rivière des Remparts; RDC = Rond Des Chevrons.

The likelihood comparison of the seven models (M1–M7) tested with *fastsimcoal2* (Supplementary Fig. S5) revealed that models including gene flow (M3, M4, M7) had a better fit than those assuming only population size changes (Fig. 3), suggesting that both events are essential to explain the evolutionary history of the Mascarene petrel. The lowest ΔAIC (difference in Akaike Information Criteria (AIC) to the best demographic model) values were observed for the *bottleneck with gene flow* model (M3) and the *ancient & recent bottlenecks with gene flow* model (M7). The model M3 assumed that the fragmentation of the ancestral population into RDC and RIR was followed by a population decline and a reduction in population connectivity between the two colonies. The model M7 parallelized the previous model but included an ancient population decline preceding the fragmentation of the ancestral population into RIR and RDC. Both models exhibited: a similar AIC (AIC = 42,029.1 and 42,031.3; Supplementary Table S2), overlapped on the log_10_ likelihood distributions across the 100 independent simulations performed per model (Fig. 3), a good fit between the observed and expected 2d-SFS (Supplementary Fig. S6), which combined suggest a similar model fit. Similar to the *Stairway Plot,* the parameters estimated by M7 suggested a large ancestral population (ca. 4 × 10^5^ birds) that suffered a significant reduction in size ca. 37 kyr (< 1000 birds). The population remained small for many generations, and ca. 1.5 kyr became structured (i.e., split) into RDC and RIR. The colonies remained connected after the population split, but exchanged few migrants (2_Nm1_ = 0.5, where 2_Nm_ is the average number of haploid immigrants entering the population per generation). This event was followed by a population decline during the past 100 years (i.e., ≤ 4 generations) that reduced the RDC and RIR populations to less than 100 breeding pairs each, and resulted in the almost complete isolation of the colonies (2_Nm0_ = 0.01; and only from RDC to RIR) (Fig. 2b). Although the 95% confidence intervals (CI) for M7 reflect uncertainty in the parameter estimates (but see 95% CI for M3; Supplementary Table S4), all parameters inferred by the seven models fall within the same orders of magnitude and were consistent with (i) a very large ancestral population (≥ 2.6 × 10^5^ birds); (ii) a population split during the last two millennia; (iii) a population bottleneck for RIR and RDC less than 100 years ago; (iv) and a very low contemporaneous population size (Supplementary Table S3).

## Discussion

In this study, we used a genome-wide SNPs dataset and two complementary modeling approaches to investigate how the past demographic events of the critically endangered Mascarene petrel were affected by natural vs. anthropogenic changes in Réunion Island. Our results reveal that (i) the known Mascarene petrel colonies exhibited a small population size and were isolated long before human colonization; (ii) historical population fluctuations coincided with significant volcanic events at Piton des Neiges and Piton de la Fournaise, unrelated to past climatic events; and (iii) the increasing human impact over the last century exacerbated the population bottlenecks further, driving the species perilously close to extinction. To our knowledge, this study provides the first long-term insights about the main drivers of biodiversity loss in an island endemic, and suggest that the current conservation status of endemic species may be a legacy of historical volcanism, exacerbated by recent human impact.

### Fine-scale population structure and historical gene flow

The F_ST_ estimates and clustering analyses confirmed that Mascarene petrel colonies constitute two genetically differentiated populations. The physical isolation of breeding populations and philopatry are recognized as one of the main barriers to gene flow in seabirds^40,54^. The Mascarene petrel colonies are separated by only 19 km straight-line distance, and ecological barriers to gene flow are unlikely as there is no marked terrestrial ecological gradient between them. Fine-scale population genetic structure was also reported for the endemic Barau’s petrel, despite the studied colonies being only 5 km apart^55^, and in other procellariforms (e.g., *Pelecanoides garnotii*)^49^. As for Barau’s petrel, our findings suggest that the most likely mechanism promoting genetic isolation between the two colonies is natal philopatry (i.e., the behavior of returning to the natal colony to breed)^56^. Although this behavior is still not well understood, the familiarity with the breeding area might increase an individual’s probability of finding suitable nesting habitat and a mate. Dispersal to a new colony could reduce inbreeding and competition, but would bring additional risks such as the time cost of searching an alternative breeding site and the higher potential to encounter unforeseen threats (e.g., predators)^56–58^. It is plausible that philopatry evolved by favoring individuals that returned to their natal site, while those individuals that attempted to find a new breeding site experienced higher mortality and/or lower reproductive success^56^. Philopatry is often associated with other isolating mechanisms^59^. Morphometric analyses suggested that birds from RIR and RDC are phenotypically indistinguishable (data not shown), but differences in breeding phenology were observed (i.e., egg laying started in October in RIR and from November to December in RDC)^42^.

Despite the strong philopatric behavior of the Mascarene petrel, our results indicate historical genomic admixture among colonies and three lines of evidence supports this. First, the three best-ranked demographic models with *fastsimcoal2* assumed that populations were more connected in the recent past than in present times. The comparison of the models M2 and M3, which only differ on the migration matrix, confirmed that the evolutionary history of the Mascarene petrel cannot be explained without historical gene flow. Indeed, all models tested with *fastsimcoal2* agree that colonies were connected until relatively recently (< 2 kyr). Second, capture-mark-recapture data (n = 42 adult birds for RDC and 37 for RIR) confirmed that birds from a given colony have never been re-sighted as a breeder or prospector at the other colony during the last four and six breeding seasons for RDC and RIR, respectively^60^, therefore one could reject the hypothesis of recent gene flow. Third, the relatedness analyses confirm the capture-recapture data by showing that birds are not reproducing outside of their known colony (i.e., birds were related only to birds sampled at their own colony; Supplementary Text S1). The absence of first, second- or third-degree relatives outside their breeding colony confirms the absence of gene flow in recent generations.

Clustering methods also highlighted six individuals from RIR with an assignment probability to RDC ≥ 50%. It is plausible that birds moved from RDC to RIR, as the demographic analyses systematically suggested a larger N_e_ for RDC (= source population) in the recent past. Alternatively, migrants may be a legacy of birds that lost their breeding nests at the time of the ancestral population split, and subsequently moved to the nearest colony to breed (RIR; see below for details). Finally, clustering analyses revealed that all light-grounded birds originated from RDC. These results are not surprising as there is no significant light pollution in Rivière des Remparts canyon, except when fledglings reach the city of Saint-Joseph (Fig. 1a). In contrast, birds from RDC face light pollution while crossing the island towards the sea^41^. Over the last two decades, a yearly rescue program based on large-scale public awareness led by a local NGO (Society of Ornithological Studies of Réunion, SEOR), has successfully rescued and released 58 Mascarene petrels disoriented by artificial lights (1996–2021)^38,41^. While the rescue campaign stands out as the most impactful measure to mitigate light-induced mortality, complementary actions such as installing light shields, employing motion sensors to turn lights off, and implementing light restriction during the peak fledging period (recently implemented in Réunion Island) could also be effective in the conservation of the Mascarene petrel^61^. Altogether, our results suggest that birds from both colonies were part of a larger population that was connected until a recent past, and genetic differentiation of populations must have been a relatively quick evolutionary event, likely exacerbated by high natal philopatry.

### Volcanic activity and recent anthropogenic impact on endemic seabird

The coalescent framework revealed two population declines of the Mascarene petrel pre-dating human impact on Réunion Island. According to the *Stairway Plot*, the Mascarene petrel reached its population maximum ca. 1 Mya and remained large until the Last Interglacial (LIG; 132–112 kyr). Given the recent origin of Réunion Island^15^, such a large population size may correspond to the spatial and demographic expansion of the species after its founding event on Réunion Island. Both methods implemented in our study identify a very large ancestral population, suggesting that favorable breeding habitat for the Mascarene petrel were abundant. The Mascarene petrel subsequently underwent a drastic population decline that started ca. 125 kyr. This date period does not coincide with any major climatic changes (Fig. 2c). Instead, it corresponds to a period of major eruptions of Piton des Neiges ca. 137–70 kyr^62^, with massive destructive impacts on terrestrial ecosystems (Fig. 2d). During this period, a large volcanic edifice (Piton des Neiges phase 4; PN4) appeared in the topographic paleo-depressions incised in the central part of Piton des Neiges by erosion during the preceding (ca. 40 kyr) period of eruption quiescence^63^. The successive volcanic eruptions fed lava flows that progressively accumulated over a thickness > 500 m in the paleo-topographic depressions. Lava flows are known to be particularly destructive at ground level, by burning all vegetation^64^. In Réunion Island, wildfire propagates in the valley scarps when the lava reaches their base, as observed during the August 2015 eruption of Piton de la Fournaise^65^. It is therefore likely that the flora and fauna on the scarps of the topographic paleo-depressions filled by the lava flows of PN4 were recurrently impacted by such wildfires and the emitted volcanic gas.

The parameters estimated by the *fastsimcoal2* models M3 and M7revealed an identical demographic history for the last two millennia, but M7 allows us to delve further into the past and suggests a significant reduction in population size ca. 37 kyr, which parallels a previously documented population decline of *Coffea mauritiana* in Réunion Island^29^, and could be related to the last explosive activity of Piton des Neiges (Fig. 2d). Although confidence intervals reflect uncertainty in the timing of this event, a substantial N_e_ reduction was also suggested by the *Stairway Plot.* From ca. 40–27 kyr, Piton des Neiges experienced successive explosive eruptions that fed pyroclastic flows (fast moving fluidized mixture of hot lava blocks, ashes and volcanic gas) on the volcano flanks and the deep valleys^17^. Moreover, this eruptive period was characterized by a major eruption dated at 37.2 ± 1.5 kyr that spread a 30–40 cm thick fallout deposit over the flanks of Piton des Neiges and the western part of Piton de la Fournaise suggesting a massive island-scale impact on most components of the island terrestrial biodiversity^66,67^. Thus, the volcanic eruptions of Piton des Neiges may have triggered Mascarene petrel population declines, through a high mortality, breeding failures and, most importantly, the total disappearance of suitable breeding habitat^11,13^. Volcanic-related population declines and extinctions have also been reported for other birds’ species on other islands. For instance, the volcanic activity at Deception Island resulted in the near-complete local extinction of the gentoo penguin colony on Ardley Island (Antarctic Peninsula)^12^; the eruption of the Barcena volcano in San Benedicto (Revillagigedo Archipelago) resulted in the local extirpation of the Townsend’s shearwater^68^, and the recent violent eruption of Kasatochi volcano (central Aleutian archipelago) caused the local extinction of avian biodiversity^13^. After the latest eruption of Piton des Neiges abruptly ended, the Mascarene petrel appears not to have recovered to previous population levels. Two types of geological events may have limited population recovery. First, the current deep valleys cutting the southern flank of Piton des Neiges started to incise PN4 around 30 kyr^69^. In Réunion Island, the intense and ongoing erosion results from river incision and valley scarp collapses^70,71^. Such scarp collapse events could have limited the colony development. Second, the volcanic zone located between the summits of Piton des Neiges and Piton de la Fournaise experienced several eruptions during the past 30 kyr, feeding lava flows that cascaded down the Bras de la Plaine valley that currently hosts the RDC colony^72,73^. The two ancient population declines for the Mascarene petrel are corroborated by a previous study considering a dataset of 22 light-grounded birds and using microsatellite loci^30^.

Although the *Stairway Plot* method does not allow us to infer the population dynamics of the Mascarene petrel during the recent past, the *fastsimcoal2* M7 suggests that during the last two millennia (ca. 1.5 kyr), the Mascarene petrel population was fragmented into two genetically distinct clusters found at the two known colonies (RIR and RDC), with reduced levels of post-split gene flow observed among them. This split follows one of the largest flank eruptions of Piton de La Fournaise ca. 1.9 kyr^66^ (Fig. 2d), starting on the upstream bank of Rivière des Remparts valley (Cratère Commerson). This eruption fed a large lava flow that entirely covered the valley floor down to the ocean, 21 km downstream. Similar to the PN4 period of activity of Piton de Neiges, this major volcanic event may have burned the vegetation on the cliffs hosting the Mascarene petrel nest sites. Consequently, many petrels may have been either fatally injured or displaced. Even if birds had survived this event, the philopatry behavior and nest-site fidelity of petrel species imply that breeders continually return to the same place every year^74^, even if breeding habitats are no longer available. For instance, the closely related Tahiti petrel (*Pseudobulweria rostrata*) nests in nickel-rich areas in New Caledonia. It was recently shown that after 10 years of nickel-mining exploration, Tahiti Petrel vocal activity was still recorded at the breeding sites, suggesting that former breeders whose nests sites were destroyed by mining activities were still visiting the area but failed to reproduce^75^. Likewise, it is plausible that many of the Mascarene petrels that were displaced by the volcanic event at ca. 1.9 kry BP did not move to another breeding site.

Once fragmented, both Mascarene petrel colonies underwent a synchronous population bottleneck during the last century, and the species was even subsequently considered extinct^37^. This population decline coincided with human-mediated changes in the landscape. Since human settlement ca. 400 years ago, human impact was rapid and substantial. Major bird predators were introduced shortly after human arrival (*Rattus rattus* in 1675; *Rattus norvegicus* in 1735, and cats in 1703)^5,76^. Extensive deforestation for logging, agriculture and cities started 200 years ago and resulted in the destruction of 70% of the native vegetation^77^. This had a significant impact through forest clearing and the spread of invasive mammals, the latter leading to increased egg and chick predation^4,74^. More recently, light pollution increased rapidly on the island, with the extension of urban and industrial areas (Fig. 2e), resulting in mortality of petrels and shearwaters, including the Mascarene Petrel^36,41^. These anthropogenic impacts are not unique to Réunion Island and human-related population collapses/extinctions are well-documented for Procellariform species, including the *Hydrobates leucorhous* on Grand Colombier Island^4^, the *Pseudobulweria rupinarum* on Saint Helena Island^74^, *Puffinus olsoni* on the Canary Islands^78^, and *Puffinus boydi* on Bermuda^79^. Current anthropogenic impacts may be compounded by the ongoing loss of nesting habitat via erosion, as illustrated by the recent collapse of Bras de Mahavel^70^.

### Conservation status of Mascarene petrel is not a legacy of climatic changes

Quaternary climatic cycles and subsequent sea-level oscillations have been invoked as one of the major drivers of avian diversification^26,53,80^, including seabird species^7,81–83^, as they depend on marine ecosystems for foraging and on terrestrial habitats for reproduction. Our study suggests that the demographic dynamics of the Mascarene petrel do not follow the historical climatic events that took place in the WIO (Fig. 2c). Paleoenvironmental records available from Lake Tritrivakely in the Central Highlands of Madagascar^84^ and Last Interglacial corals on the Seychelles Islands^85,86^ confirmed that the LIG was characterized by warm temperatures and by higher-than-present sea level. This would be in line with a larger population size for the Mascarene petrel for this period, as its marine distribution seems to be related to high sea surface temperatures. However, the Sta*irway Plot* analyses revealed a large decline ca. 125 kyr that could not be explained by climatic conditions alone, but coincided with volcanic activity of Piton des Neiges (see discussion above). There is multiple evidence that the Last Glacial Maximum (LGM; 26.5–19 kyr) was colder and drier across the WIO, with a substantial decrease in the annual mean temperature (ca. − 4 °C than in the present days), a reduction in rainfall (ca. − 10%), a reduction in sea-level (ca. − 125 m than today), and in the likely reduction of forest habitats^84,87^. Assuming that the Mascarene petrel population dynamics were influenced by climatic shifts, the population decline would be expected to coincide with the onset of the LGM, but both coalescent approaches agree that the decline pre-dated this period (ca. 125 and 37 kyr). The demographic analyses also suggest that Mascarene petrel never recovered its population size, not even during the African Humid Period (AHP; 15–5 kyr), a period characterized by an abrupt warming and increasing levels of moisture in continental Africa^88,89^, Madagascar^9,90^ and Rodrigues^19^, and that was followed by several megadroughts. The absence of population recovery could, instead, be explained by the persistent reduction in breeding areas for the Mascarene petrel and mortality following intense volcanic activity on Réunion Island, compounded by philopatry and mating behavior (i.e., monogamy) of the species. Altogether our study suggests volcanism as the major force driving the ancient population dynamics of the Mascarene petrel, but we emphasize that molecular chronologies are dependent on appropriate molecular rates^8,9,26^. The present study used the generation time of the Barau’s petrel for time calibration, but a different generation time estimate may result in older or more recent demographic events. We also highlight that the *Stairway Plot* method assumes that the observed data stems from a panmictic population^43^ and deviations from these model assumptions, such as the existence of migration among sub-populations, may bias demographic interpretations^27,33,34,91^. Moreover, it has also been demonstrated that N_e_ estimates are sensitive to the number of loci used in the analyses^47,50^, even though the general trend of population size change over time remains unaffected^10^. Consequently, the absolute N_e_ estimates were interpreted with caution.

One of the most striking results of our study is the lower population size and fragmentation prior to human impact on Réunion Island. Both coalescent approaches implemented in this study show that at the time of the first human settlement, the Mascarene petrel had an increased risk of extinction (N_e_ < 1000 birds), but the cumulative human-impact likely reduced the effective population size further. It is plausible that past volcanism also had catastrophic impacts on other endemic species, especially for those that are restricted to small ranges and/or distributed near the volcanic massifs, such as is the endemic Barau's petrel. Hence, multi-taxa studies are necessary to address the impact of volcanism on Réunion Island and better understand historic and current drivers of biodiversity loss. To conclude, our study suggests that historic volcanic activity may be the primary trigger of the current conservation status of endemic island biodiversity. The past dramatic population fluctuations combined with current anthropogenic impacts ultimately downward species population size to a minima, hampering its recovery from population lows and driving species towards extinction.

## Materials and methods

### Study species

The Mascarene petrel is the only representative of the *Pseudobulweria* genus in the Indian Ocean^36,38,42^. The species is endemic to Réunion Island and was thought to be extinct for more than eight decades, until it was rediscovered in the twentieth century with a dead grounded bird^37^. Nevertheless, the two breeding colonies investigated in this study (RDC and RIR; Fig. 1a) were only discovered in 2016–2017^38^. The biology of Mascarene petrel remains poorly known as birds spend most of their life at sea and their breeding grounds are restricted to remote and inaccessible cliffs^38^. Currently, the population size is estimated to be as low as 100 breeding pairs^42^ and the petrel is listed as Critically Endangered (CR, IUCN 2020). Conservation actions focused on Mascarene petrel effectively started in the last two decades with the implementation of a rescuing program for light-grounded birds^41^ and predator control at breeding colonies^38^.

### Study area and specimen collection

The mountainous Réunion Island is dominated by high, steep reliefs resulting from successive periods of intense volcanic activity and high erosion rates^92^. The highest relief reach 3071 m a.s.l. and 2631 m a.s.l. at Piton des Neiges’ and Piton de la Fournaise’s summits, respectively (Fig. 1a). The climate is characterized by a thermal gradient associated with elevation, with an annual mean temperature ranging from 25 °C on the coast to 10 °C at the summits. High mean annual rainfall is recorded on the windward east side of the island (ca. 11,000 mm), whereas the climate is markedly drier on the leeward west side (ca. 500 mm)^77^. Urban areas are mostly located on the coastal lowlands (< 1300 m a.s.l.), while the native forest is restricted to intermediary and high elevations^6,29,77^. Mascarene petrel breeding grounds are located in the upper valleys of large rivers, i.e., in the vicinity of past or dormant volcanic areas (Fig. 1a). The first site, RDC (− 21.16°N, 55.51°E), lies in the Bras de la Plaine valley (1250 m above sea level, a.s.l.), and covers an area of about 10,000 m^2^. The second site, RIR (− 21.29°N, 55.61°E), is located in the Rivière des Remparts valley (650 m a.s.l.) and covers a smaller area (~ 800 m^2^)^38^. All samples used in this study come from wild animals. Birds were captured by hand at their nesting burrows between 2016 and 2018. Blood samples (approx. 0.5 ml) were collected by venipuncture of the medial metatarsal or basilic veins. Bird capture, handling and sample collection were approved and carried out in concordance with the principles of the Research center on biology of bird populations (PP 609 and banding authorization 44 of MLC; CRPBO, National Museum of Natural History, Paris) and approved by Réunion Island National Park. All birds were released at their burrow after sampling. All sections of this manuscript followed the ARRIVE guidelines^93^ for reporting animal research. Blood samples of grounded birds (attracted by light pollution), were collected with the same method between 2008 and 2018 by the SEOR. All samples were stored in 70% ethanol until DNA extraction. Genomic DNA was extracted using the QIAmp Blood and Tissue kit (Qiagen). Collection information for all specimens is given in Supplementary Table S1.

### SNP genotyping and filtering

SNP genotyping was carried out by Diversity Arrays Technology (DarT Pty Ltd, Canberra) using the DArTseq™ protocol, a restriction site-associated DNA sequencing method similar to double‐digest restriction‐associated DNA sequencing (ddRAD)^24,94^. A DArT library was prepared using 20–40 ng/μl of DNA and the restriction enzymes *PstI* and *SphI*, following the protocols described by Georges et al. (2018). Sequence data was processed using a proprietary DArT analytical pipeline^95–97^. In the absence of a conspecific reference genome, loci were aligned to the genome assembly of *Calonectris borealis*^39^ (family Procellariidae; GCA_013401115.1; Coverage depth = 130×)^98^. Given the high genomic synteny within birds, the overall genomic structure and gene order is expected to be very similar between members of the same family^99^. Such synteny has been used for SNP mapping in other seabird studies^99,100^. The raw SNP data were filtered in-house using the *dartR* v 2.1.4^101^ *R* package (*R* v4.2.1). Loci for which SNPs has been trimmed from the sequence tag along with the adaptor were filtered out. Genome-wide SNP datasets may contain large numbers of linked loci, which can break assumptions of independence for many analyses^24^. To control for short-distance linkage disequilibrium, we filtered out multiple-linked SNPs per sequenced tag (i.e., only one SNP per fragmented was retained at random). Knowing also that individuals with a low call rate (i.e., proportion of scored loci for an individual) and high heterozygosity may indicate bad DNA quality or cross-sample contamination^102^, individuals with more than 20% of missing data^103^ and/or with a very high individual heterozygosity (≥ 13% for Mascarene petrel; see Supplementary Fig. S7) were removed from our dataset. Any monomorphic loci (i.e., SNP fixed over all individuals) arising as a result of the removal of individuals were also deleted. Further filtering was undertaken for SNP with a call rate (i.e., proportion of scored individuals for a locus) lower than 90% and a genotyping reproducibility (i.e., average repeatability of alleles at a locus across replicates) below 95%. Loci with an average read depth (i.e., counts of sequence tags scored at a locus) lower than 10×^25,29,45,46^ and higher than 40×, and a minor allele frequency (MAF) lower than 0.01 were also removed^104^. The SNP dataset was then tested for the presence of sex-linked markers, knowing that if a SNP is present only on the Z chromosome but not on the W chromosome, all females (ZW) will be heterozygous at that locus and all males (ZZ) homozygous. Sex-linked markers were removed from the dataset. Finally, outlier SNPs were identified using two algorithms: *PCAdapt*^105^ and *OutFLANK*^106^*,* using a cut-off value of 0.01^102^. Significant outliers were then excluded as they may represent loci under selection. All thresholds were defined after plotting data ^102,104^. The filtered dataset contained a total of 13,855 SNP loci and 87 birds (dataset 1). After population clustering analyses, loci that exhibited departures from Hardy–Weinberg equilibrium (HWE) were also excluded (12,784 SNP loci; 87 birds; dataset 2). Information about the number of SNP loci and individuals retained at each step of the quality-filtering analyses are given in Supplementary Table S5.

### Population genetic structure and relatedness analyses

The coalescent framework makes several assumptions about the population from which the samples are drawn^22^. Multiple studies have shown that population structure (i.e., non-random mating)^32–35,107^ and relatedness^102^ between individuals can confound genomic inferences. To minimize the violation of these assumptions, and to examine philopatric characteristics of the species, the population genetic structure of Mascarene petrel and relatedness between the 87 birds that passed the SNP quality filtering (dataset 1) were investigated first. The population structure was examined using three different approaches. First, a dimensionality-reduction clustering was performed with Discriminant Analysis of Principal Components (*DAPC*), using the *Adegenet* v2.1.7^108^ *R* package. *DAPC* summarizes the information present on the individuals’ genotypes using principal components analysis prior to a discriminant analysis to maximize differentiation between groups while minimizing variation within groups and makes no assumptions about HWE or linkage disequilibrium^109^. A K-means clustering algorithm was employed to identify the optimal number of clusters from K = 1 to K = 5. The optimal number of clusters (K) was determined by the lowest Bayesian Information Criterion (BIC) following^109^. To avoid overfitting of discriminant functions, a-score optimization was used to evaluate the optimal number of principal components to retain in the analysis^45^. Second, the Bayesian assignment approach implemented in *Structure* v2.3.4^110^ was used to assign individuals to a specific number of clusters (K). The number of genetic clusters ranged between 1 and 5, and a total of 10 independent runs were performed for each value of K to check the consistency of results. Analyses were performed assuming an admixture model, correlated allele frequencies, and without population priors, for 10,000 burn‐in and 100,000 replicate runs. Two criteria were used to determine the optimal number of genetic clusters: the log likelihood given K (L(K))^110^, and the second-order rate of change of mean log likelihood (ΔK) following the Evanno’s method^111^. Both L(K) and ΔK were calculated using *Clumpak*^112^. Third, genetic differentiation between the genetic clusters previously detected by the *DAPC* and *Structure* analyses was estimated using Wright’s F-statistics F_ST_^113^, with and without the light-grounded birds. The analyses were performed with *dartR* v 2.1.4^101^ *R* package. Significance was tested using 10,000 bootstraps.

The genetic relationship between two individuals can be described by the concept of identity-by-descent (IBD; i.e., two alleles are identical if they share a recent common ancestry)^114^. To account for the potential presence of related individuals in our dataset, the software *ngsRelateV2*^114^ implemented in *ANGSD* framework^115^ was used to estimate the nine condensed Jacquard coefficients between all pairs of individuals^116^ (see Supplementary Text S2 for details). These coefficients provide a comprehensive description of the common ancestry between two individuals and were used to infer their familial relationship^114,117^. Only one individual out of a group of closely related individuals (e.g., parent–offspring or full siblings) was retained in dataset 3 (60 individuals; 12,731 SNPs). See Supplementary Text S1 and Fig. S8 for details about SNP datasets used for each analysis.

### Mutation rate and generation time

Currently, few mutation rate and generation time estimates are available for Procellariiformes, hence demographic analyses for the Mascarene petrel were performed using the mutation rate of the Northern fulmar (*Fulmarus glacialis*; 2.89 × 10^−9^ substitutions per nucleotide per generation)^53^. This mutation rate is the only one currently available for Procellariiformes and has been used for similar analyses in other species from the same family, e.g., *Puffinus mauretanicus*^118^*.* Due to the recent rediscovery of the Mascarene petrel, some demographic parameters requiring long-term monitoring of marked birds (especially prebreeding survival rate and age at first breeding) are still missing, precluding the estimation of its generation time (GT). However, the equivalent parameters were available for the endemic Barau's petrel (*Pterodroma baraui*) and these were used to estimate GT (18.9 years; see Supplementary Text S3 for details about GT estimation for Barau's petrel) by the authors. As Mascarene and Barau's petrel are of similar size and both are exposed to similar environments and anthropogenic threats, the generation time estimated for Barau's petrel was used in this study.

### Demographic history of Mascarene petrel

#### Stairway plot

The genome-wide distribution of allele frequencies of a given set of SNPs in a population (i.e., Site Frequency Spectrum; SFS) has been widely used as a summary statistic for demographic inferences^119^. The *Stairway plot* method is a model-free approach that makes use of the SFS generated from population genomic data to estimate a series of population mutation rates (θ = 4N_e_μ), following a multi-epoch demographic model where epochs coincide with coalescent events^119^. The genlight SNPs dataset without sites in HWE departure (dataset 2; 87 individuals; 12,784 SNPs) was initially converted to a VCF (Variant Call Format) file using the *gl2vcf* function implemented in *dartR* v 2.1.4^101^ *R* package. The VCF file was then used to estimate the 1d-SFS (i.e., the SFS for a single population) using the *easySFS* tool (https://github.com/isaacovercast/easySFS)^119,120^. Analyses were performed for each genetic cluster (i.e., RDC and light-grounded birds; and RIR) detected by the population structure analyses, to satisfy the assumption of population panmixia^22^. In the absence of a suitable outgroup to determine the ancestral state of each allele, we considered the minor allele-frequency spectrum (i.e., MAF or folded SFS) for all SFS-related analyses^119^. MAF selects the least frequent of the two alleles within the dataset and uses that information in the summary SFS. Since the SFS cannot be computed for sites containing missing data, the number of individuals per population was projected down to recover a higher number of loci^120^ (n = 45 for RDC and light-grounded birds; and n = 33 for RIR). The resulting 1d-SFS was used as input data to generate 199 bootstrap replicates to obtain 95% confidence intervals, using the script provided by *Stairway plot v2.0*^119^*.* Inferences were finally made based on 200 SFS with *Stairway plot v2.0 0*^119^*.* Since this method is sensitive to the number of coalescent events^47,50,121^, analyses were initially performed considering the entire datasets, and repeated with an equal sample size (n = 33; individuals randomly selected using *R*) to avoid differences in N_e_ among populations related to sample size. To evaluate the impact of relatedness on demographic inferences of Mascarene petrel, the *Stairway Plot* analyses were also repeated considering the dataset without closely-related individuals (n = 35 for RDC and light-grounded birds; and n = 19 for RIR after downsampling). Finally, the analyses were repeated for the entire dataset (n = 54 after downsampling and considering only unrelated individuals). Plots were scaled using 2.89 × 10^−9^ substitutions per nucleotide per generation^53^, and 18.9 years as generation time.

### Demographic modeling with *fastsimcoal2*

The composite-likelihood approach implemented in *fastsimcoal2*^44^ allows the inference of demographic parameters from the joint allele-frequency spectrum (JAFS) of populations using a coalescent simulation framework. A total of seven demographic models were tested for the Mascarene petrel, following a 3-step approach. First, four demographic models assuming size changes after the fragmentation of the ancestral population into the two known colonies, RDC and RIR, were tested. Specifically, the first and simplest model assumed a population split (with population resize) into RDC and RIR colonies (*null model*, M1). The second model tested the occurrence of a population decline for both colonies after population split and assumed that colonies have been isolated since then (*bottlenecks without gene flow*, M2). The third model also relied on the occurrence of a population decline for both colonies after population split, but assumed that colonies were exchanging migrants (i.e., 2_Nm1_ = 0.5, where 2_Nm1_ is the average number of haploid immigrants entering the population per generation). However, the decrease on N_e_ was followed by a reduction on population connectivity, with almost no gene flow (2_Nm0_ = 0.01, where 2_Nm0_ denoted recent gene flow) and migrants moving exclusively from RDC to RIR colony (= asymmetric gene flow; *bottlenecks with gene flow*, M3). The fourth model tested the occurrence of a population decline only for RIR, and a constant population size for RDC after the population split. The model also assumed connectivity changes, with the colonies becoming more isolated after the decline of RIR (2_Nm1_ = 0.5 and 2_Nm0_ = 0.01) (*asymmetric bottleneck*, M4). Second, two scenarios assuming size changes before the fragmentation of the ancestral population into RDC and RIR were tested. The fifth model (*ancient bottleneck*, M5) tested a single population decline before population split, while the sixth model (*two ancient bottlenecks*, M6) tested the occurrence of two successive declines before population split. Finally, a model including both size changes before and after the population split was built. The seventh model assumed the occurrence of an ancient population decline (i.e., before the population split) and a population bottleneck afterwards for both colonies. Similar to M3 and M4, the model implied connectivity changes, with the two colonies exchanging migrants after the population split (2_Nm1_ = 0.5), but becoming almost isolated after the declines (asymmetric gene flow; 2_Nm0_ = 0.01) (*ancient & recent bottlenecks*, M7). For the models assuming asymmetric gene flow (M3, M4 and M7), RDC was considered to be the source population as this colony covers a larger geographic area, exhibit a larger population size (as suggested by the *Stairway Plot* analyses for each genetic cluster separately), and also display lower levels of genetic admixture in comparison to RIR. See Supplementary Fig. S5 for an illustration of the seven tested demographic models, and Supplementary Table S6 for parameter tags definition and respective searching ranges. Since it was not computationally feasible to run *fastsimcoal2*^44^ for the entire dataset, a total of 20 birds (i.e., 10 unrelated individuals from each colony; dataset 4) were selected for the analyses^44,122,123^ (see Supplementary Table S1 for list of individuals). The *easySFS* tool^119,120^ was used to estimate a 2d-SFS (where the two dimensions correspond to RIR and RDC colonies) from a VCF file. Fastsimcoal2 v.2.6^44^ was run using 200,000 coalescent simulations per set of parameters and 40 ECM cycles during parameter estimation from the SFS. 100 independent runs were performed for each demographic model to determine the parameter estimates that maximize the composite-likelihoods^44^. The best-fitting demographic model was identified (i) on the basis of their Akaike’s information criterion (AIC)^124^; (ii) on the likelihood distributions obtained based on 100 expected SFS, as an overlap among the distributions between models would indicate no significant difference between the fit of alternative models as differences may be attributed to the variance in the SFS approximation^46^; and (iii) on the visual inspection of the fit between the expected and observed 2d-SFS^119^. The nonparametric block-bootstrap approach implemented in^46^ was used to generate 40 bootstrap replicates. Bootstrap replicates were obtained by dividing the SNPs into 100 blocks, and sampling with replacement 100 blocks for each replicate, to match the original dataset size^46^. A total of 40 independent *fastsimcoal2* runs were performed for each replicate under the two models with the lowest AIC. The parameter estimates with the highest likelihood from each independent run were used to estimate the 95% CI with the Rmisc R package^125^. The most realistic model was finally scaled considering a generation time of 18.9 years.

### Supplementary Information

Supplementary Information.

## Data Availability

The raw DArTseq genotypes generated by Diversity Arrays Technology (DarT Pty Ltd, Canberra) and analyzed during the current study, the associated specimen metadata, and the genomic datasets (dataset 1–4) used for all the analyses are available on DRYAD (https://doi.org/10.5061/dryad.s1rn8pkfb). Scripts are available from the corresponding author upon request.
