# Supplementary material for: Past volcanic activity predisposes an endemic threatened seabird to negative anthropogenic impacts
Source: Sci Rep. 2024 Jan 23;14:1960. doi: 10.1038/s41598-024-52556-9 (PMC10805739; doi:10.1038/s41598-024-52556-9)
Supplement: Supplementary file 1 — Supplementary Information. [file 41598_2024_52556_MOESM1_ESM.pdf]

Supplementary file for

Past volcanic activity predisposes an endemic threatened seabird to negative anthropogenic impacts

Helena Teixeira [1,*], Matthieu Le Corre [1], Laurent Michon [2,3], Malcolm A C Nicoll [4], Audrey Jaeger [1], Natacha Nikolic [5], Patrick Pinet [6], François-Xavier Couzi [7], Laurence Humeau [8]

[1] UMR ENTROPIE (Université de La Réunion, IRD, CNRS, IFREMER, Université de Nouvelle-Calédonie), 15 Avenue René Cassin, CS 92003, 97744 Saint Denis Cedex 9, Ile de La Réunion, France

[2] Université de La Réunion, Laboratoire Géosciences Réunion, 97744 Saint Denis, France

[3] Université Paris Cité, Institut de physique du globe de Paris, CNRS, 75005, Paris, France

[4] Institute of Zoology, Zoological Society of London, Regent's Park, London, NW1 4RY, UK

[5] INRAE, AQUA, ECOBIOP, France

[6] Parc National de La Réunion, Life+ Pétrels. 258 Rue de la République, 97431 Plaine des Palmistes, Réunion Island, France.

[7] Société d'Etudes Ornithologiques de La Réunion (SEOR), 13 ruelle des Orchidées, 97440, Saint André, Réunion Island, France

[8] UMR PVBMT (Université de La Réunion, CIRAD), 15 Avenue René Cassin, CS 92003, 97744 Saint Denis Cedex 9, Ile de La Réunion, France

Supplementary Text

Text S1 – Molecular datasets

A total of 93 samples were available for genomic analyses, including 32 birds from RDC, 38 from RIR, two birds from two artificial nests, and 21 light-grounded birds. The SNP filtering was performed at both individual and locus level. Of the 67,095 SNP loci called across the 93 sequenced birds with the DArTseq protocol, only 13,855 SNP passed all the quality filters and were included in our dataset (i.e., a single locus per DArT sequence; locus with a call rate ≥ 0.90; locus with reproducibility ≥ 95%; minimum read depth of 10×; Minor Allele Frequency ≤ 0.01; only autosomal loci; no outlier). Of the 93 samples sent for DArT sequencing, a total of 87 samples (31 from RDC colony, 37 from RIR colony, 19 light-grounded birds) passed the quality filters. The individuals from the artificial colony and four individuals with a high individual heterozygosity were excluded from our dataset (Fig. S7). The resulting dataset (dataset 1; 13,855 SNP loci and 87 birds) was used to investigate population structure in Mascarene petrel, and relatedness among all the 87 birds.

The relatedness analyses revealed that 60 out of 3,741 dyads were related in 1° degree (i.e., parent-offspring or full-sibling) in our dataset (data not shown). All the birds were related only to birds sampled at their own colony, or in few cases to a grounded bird (n = 3). The analyses also revealed that individuals were often related to more than one bird. Only one individual of each closely related dyad/triad/quartet partner was retained in the dataset (i.e., a total of 8 birds from RDC colony, 16 from RIR, and 3 light-grounded birds were excluded). After population structure and relatedness analyses, two new molecular datasets were created. First, loci that exhibited a departure from Hardy–Weinberg equilibrium (HWE) were excluded, but all individuals who passed the quality filters were retained (dataset 2; 12,784 SNP loci and 87 birds). Second, only individuals unrelated to other individuals of our dataset were taken (dataset 3; 12,731 SNP loci and 60 unrelated birds). Both datasets were used for the demographic analyses with *Stairway Plot*, but since the SFS cannot be computed for sites containing missing data, the number of individuals per population was projected down to recover a higher number of loci [1] during the SFS estimation (n = 45 for RDC and light-grounded birds; and n = 33 for RIR for dataset 2; and n = 35 for RDC and light-grounded birds; and n = 19 for RIR for dataset 3). A subset of dataset 3 was finally created by selecting 10 individuals from each colony for demographic modelling with *fastimcoal2* (dataset 4; 9,897 SNP loci and 20 unrelated birds). See Fig. S8 and Table S1 for details.

Text S2 – condensed Jacquard coefficients

For two diploid individuals, there are 15 possible identity-by-descent (IBD) sharing patterns at any given site (nine if we ignore the maternal or paternal origin of the alleles; review at [2]). The frequency of those nine condensed states and their corresponding frequencies in the genome of two individuals are called the condensed Jacquard coefficients (denoted from J1 – J9). In the absence of inbreeding, only three of

the condensed Jacquard coefficients can be positive (J7, J8 and J9), which are often denoted as K_2, K_1, and K_0 (i.e., probability of two individuals sharing 2, 1 or 0 alleles from a single ancestor at any locus, respectively [3].

Text S3 – Estimation of generation time for *Pterodroma baraui*

In the absence of long-term demographic data for the Mascarene petrel (*Pseudobulweria aterrima*), we estimated the generation time (GT) of Barau's petrel (*Pterodroma baraui*). Barau's petrels have been studied with mark-recapture (MR) protocols and breeding success monitoring since 2004 and 2008 at two breeding colonies (Piton des Neiges and Grand Bénare, respectively). MR data was used to estimate age at first breeding, age-specific recruitment rate, age specific annual survival, individual breeding frequency and breeding success. Using these parameters, an age-dependent post breeding matrix population model was built to explore population viability and various demographic parameters including Generation Time (GT). Matrix population modelling construction and exploration were done with the software ULM 6.0 (https://www.bio.ens.psl.eu/~legendre/ulm/ulm.html). GT was finally calculated following [4], and resulted in a mean generation time of 18.9 years for Barau's Petrel [5].

Supplementary Figures

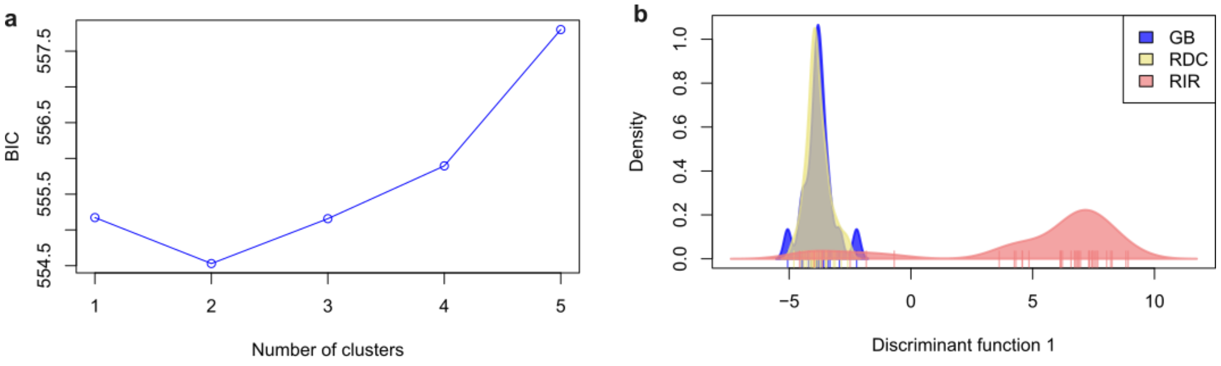

Fig. S1. Discriminant Analysis of Principal Components (*DAPC*) of genetic variation. **a,** Bayesian Information Criterion (BIC) for each K value computed using the *Adegenet* R package following (Jombart et al., 2010). Analyses suggest K = 2 as the best number of clusters for *Pseudobulweria aterrima*. **b,** *DAPC* results using 13,855 genome-wide SNPs (n = 87 birds) based on the genetic groups detected with the "*find.cluster*" function. The analyses separated the birds from RDC and grounded birds from the birds sampled at RIR. RIR = Rivière des Remparts; RDC = Rond Des Chevrons; GB = light-grounded birds.

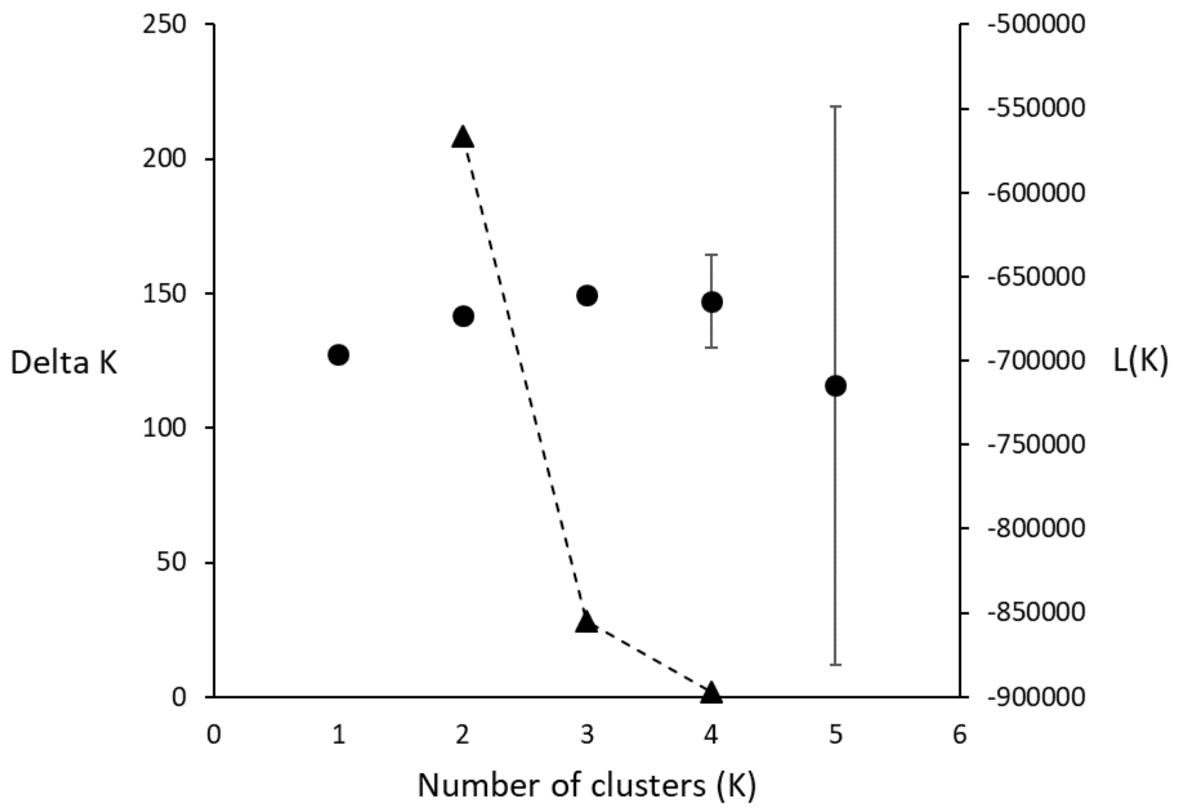

Fig. S2. Detection of the number of genetic clusters (K) using the log likelihood mean values L(K) (black circles) and Delta K statistic following the method of Evanno [7] (black triangles), as derived from *STRUCTURE* with K ranging from 1 to 5. Each value was obtained by averaging the posterior probabilities over 10 independent runs for the SNPs dataset for each K value. K = 2 is the optimal number of clusters for *Pseudobulweria aterrima*.

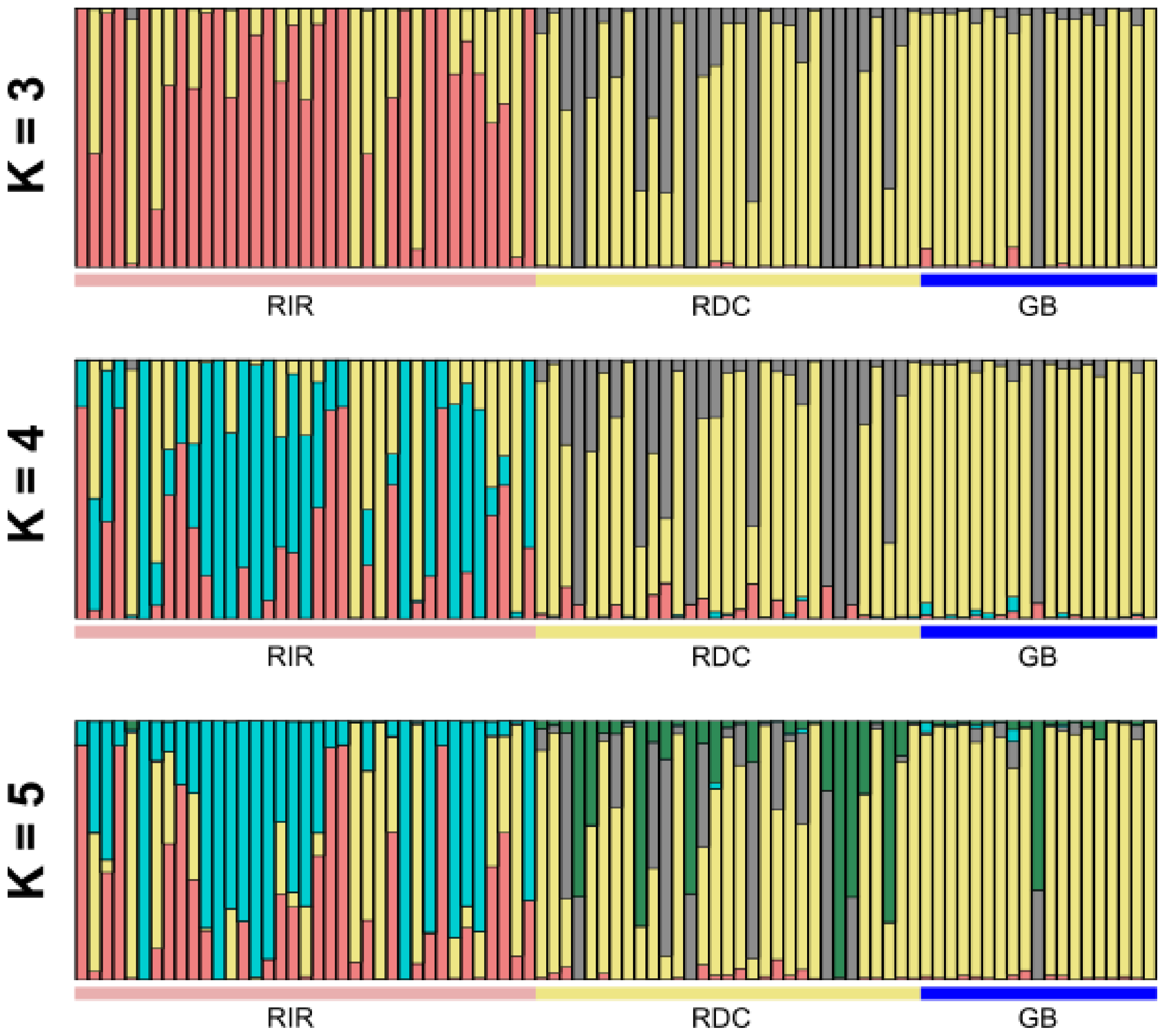

Fig. S3. Cluster assignment of 87 *Pseudobulweria aterrima* individuals to three (K = 3), four (K = 4) and five (K = 5) genetic clusters using 13,855 genome-wide SNPs with *structure* software. Each single vertical bar represents one individual and each color a distinct genetic cluster. Each value was obtained by averaging the posterior probabilities over 10 independent runs. RIR = Rivière des Remparts; RDC = Rond Des Chevrons; GB = light-grounded birds.

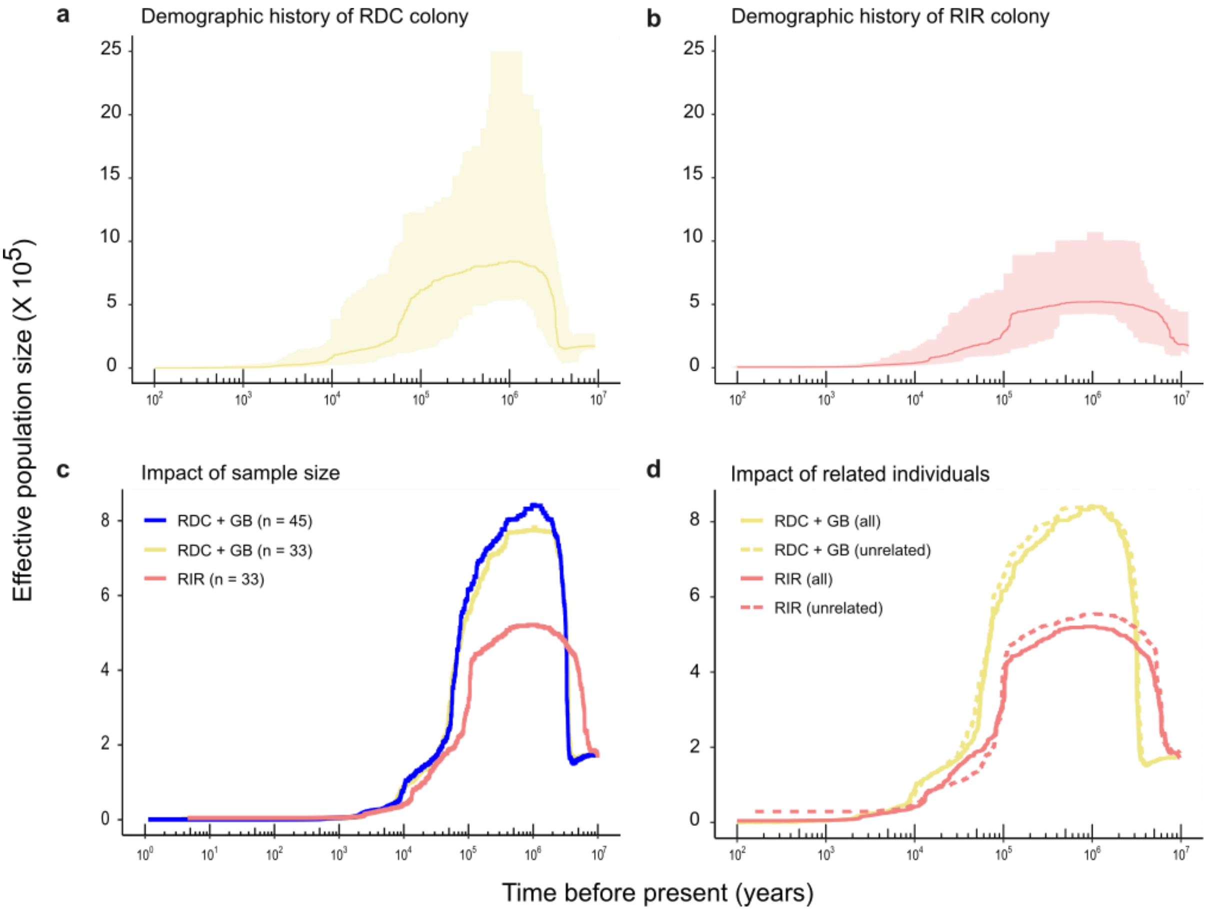

Fig. S4. Demographic history of *Pseudobulweria aterrima* inferred with *Stairway Plot* using the genome-wide SNPs dataset. **a,** Confidence interval for RDC and light-grounded birds (cluster 1, n = 45 birds) and **b,** for RIR colony (cluster 2, n = 33 birds). The thick line corresponds to the median N_e and the ribbon represents the 95% confidence interval. **c,** Comparison of the demographic history inferred for RDC and light-grounded birds (blue; n = 45) and RIR (rose; n = 33). The demographic history of RDC and light-grounded birds were repeated considering an equal sample size as for RIR (yellow, n = 33). The thick lines correspond to the median values of N_e. **d,** Impact of related individuals in *Stairway Plot* analyses. The thick lines correspond to median values of N_e estimated using all individuals (n = 45 for RDC and light-grounded birds; and 33 for RIR), and dashed lines the median N_e estimated after excluding first degree relatives (i.e., parent-offspring and full-sibs; n = 35 for RDC and light-grounded birds and 19 for RIR). None of the results showed deviations from results generated using the full dataset and assuming population structure (Fig. 2a). All analyses were performed considering 18.9 years as generation time and 2.89×10^{-9} substitutions per nucleotide per generation as mutation rate. The x-axis is plotted on a $\log_{10}$ scale. RIR = Rivière des Remparts; RDC = Rond Des Chevrons; GB = light-grounded birds.

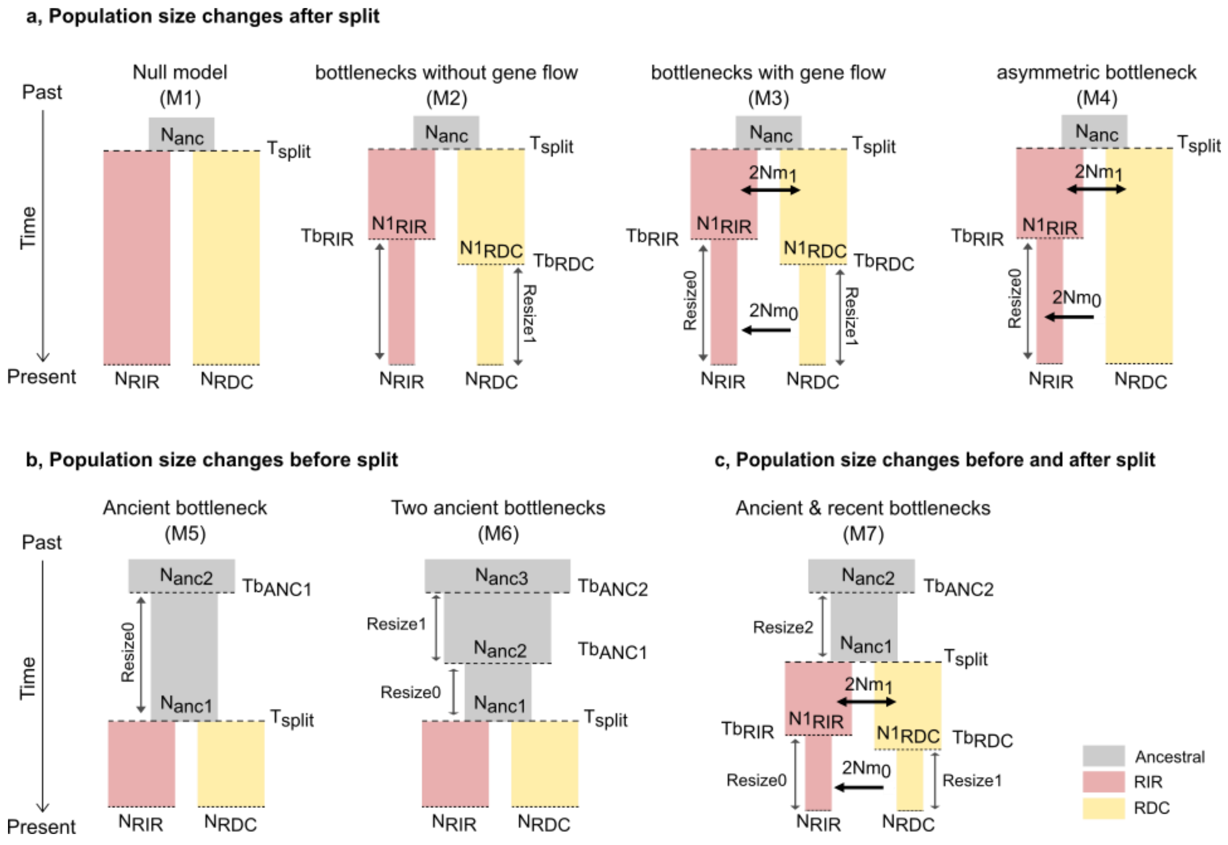

Fig. S5. Illustration of the demographic models compared with *fastsimcoal2*. Models were tested following a hierarchical approach assuming **a,** population size changes after population split (i.e., ancient population became structured into RDC and RIR colonies); **b,** population size changes before split; and **c,** population size changes before and after split. The first model assumes that an ancient panmictic population split into RDC (Rond Des Chevrons) and RIR (Rivière des Remparts) colonies (null model, M1). The second model assumes the occurrence of a population decline for both colonies after population split, without connectivity changes (bottlenecks without gene flow, M2). The third model assumes the occurrence of a population decline for both colonies after population split, but also a reduction on gene flow after the bottleneck (bottlenecks with gene flow, M3). The fourth model relies on the occurrence of a population decline only for RIR colony (asymmetric bottleneck, M4). The fifth (ancient bottleneck, M5) and sixth (two ancient bottlenecks, M6) models assume a single or two population decline before population split, respectively. The last model relies on the occurrence of a population decline before the population split and a second bottleneck afterwards (ancient & recent bottlenecks, M7). RIR is represented by rose and RDC by yellow color. See Table S6 for parameter definition and respective searching ranges. 2_{Nm} = average number of haploid immigrants entering the population per generation, where 2_{Nm0} denotes recent gene flow and 2_{Nm1} ancient gene flow among the two colonies. For M3, M4 and M7: 2_{Nm0} = 0.01 and 2_{Nm1} = 0.5.

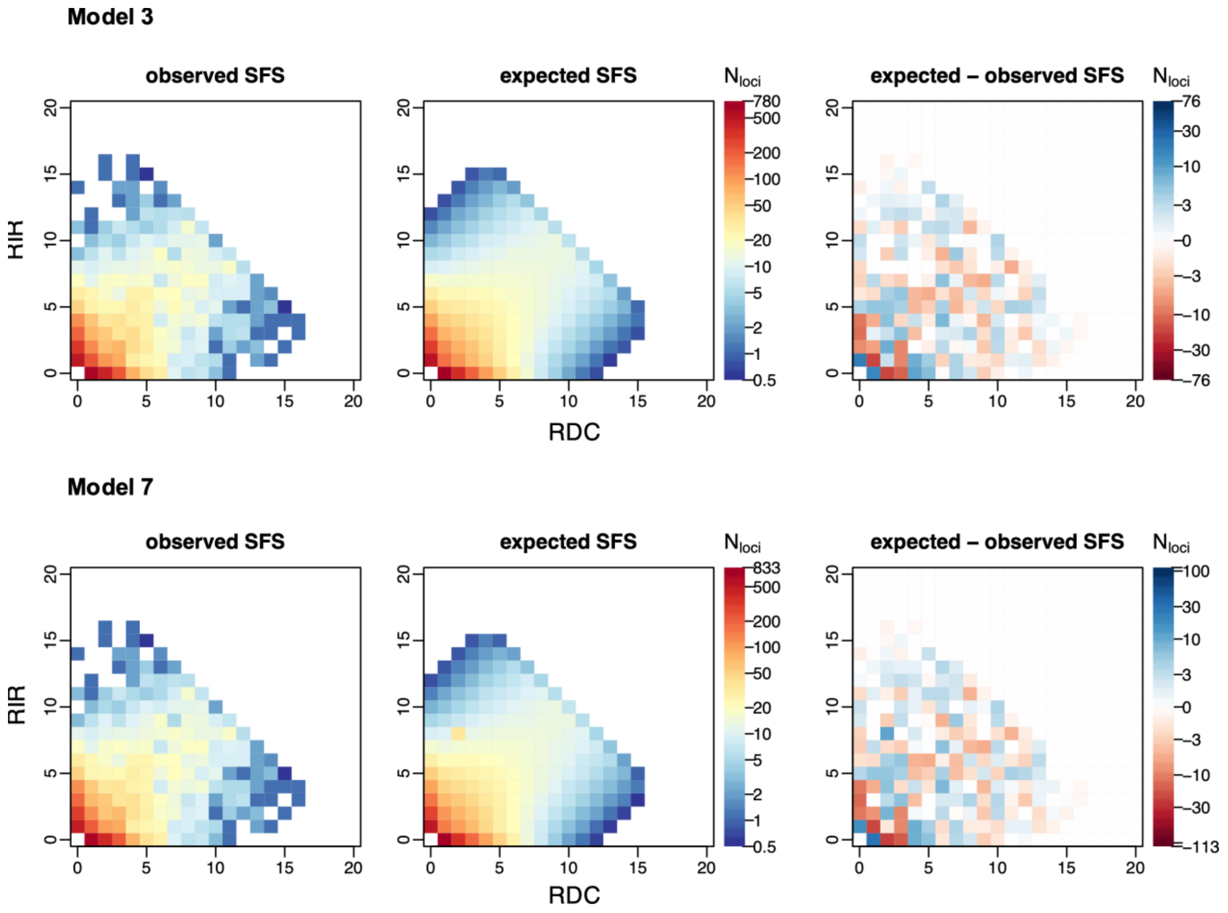

Fig. S6. Fit of the expected pairwise 2d-SFS obtained for the parameters that maximise the likelihood under the two best demographic models (M3 and M7) with *fastsimcoal2,* and the observed 2d-SFS (i.e., SFS estimated from genome-wide SNPs dataset). Each row and column show the observed or expected number of minor alleles with frequency *i* in RDC and frequency *j* in RIR, respectively. The figure shows a good fit between the two demographic models and genomic data. SFS = Site Frequency Spectrum; M3 = bottlenecks with gene flow; M7 = ancient & recent bottlenecks.

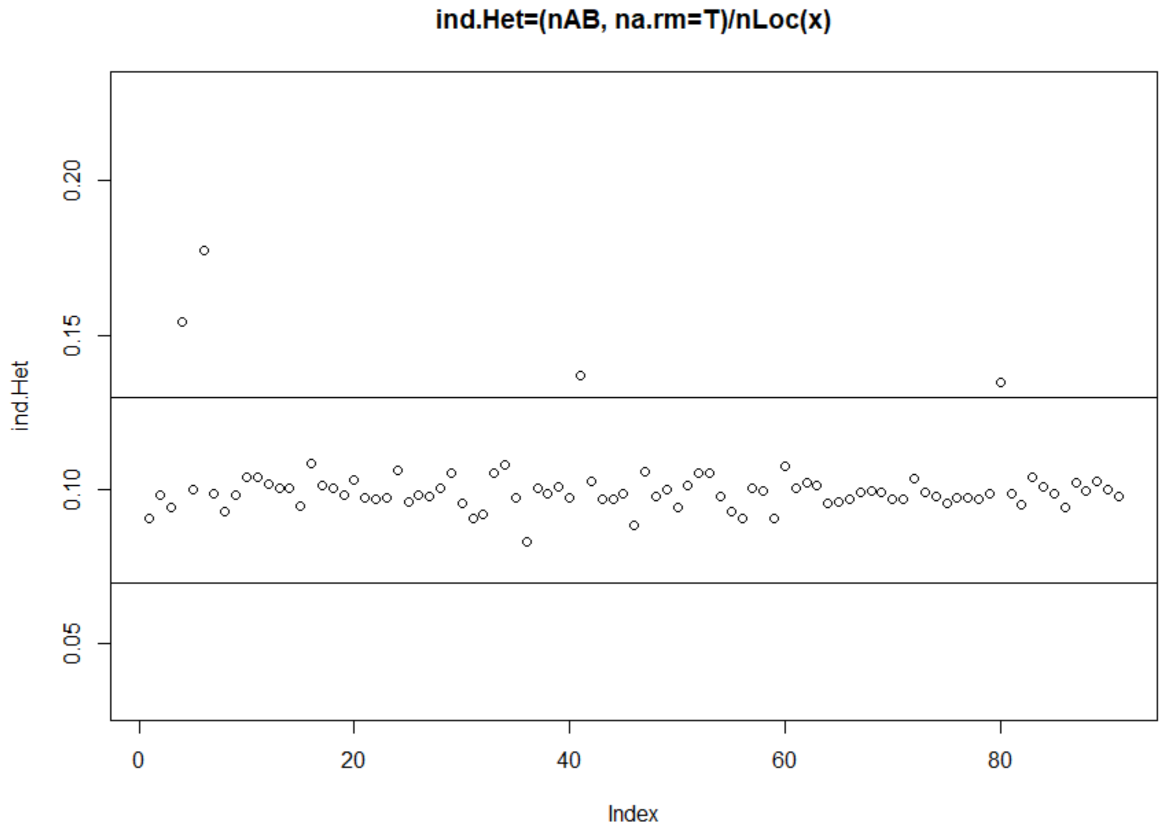

Fig. S7. Individual heterozygosity estimated following [8]. Individuals with a heterozygosity higher than 0.13 were excluded from our dataset, as a high heterozygosity could be due to cross-contamination from other samples.

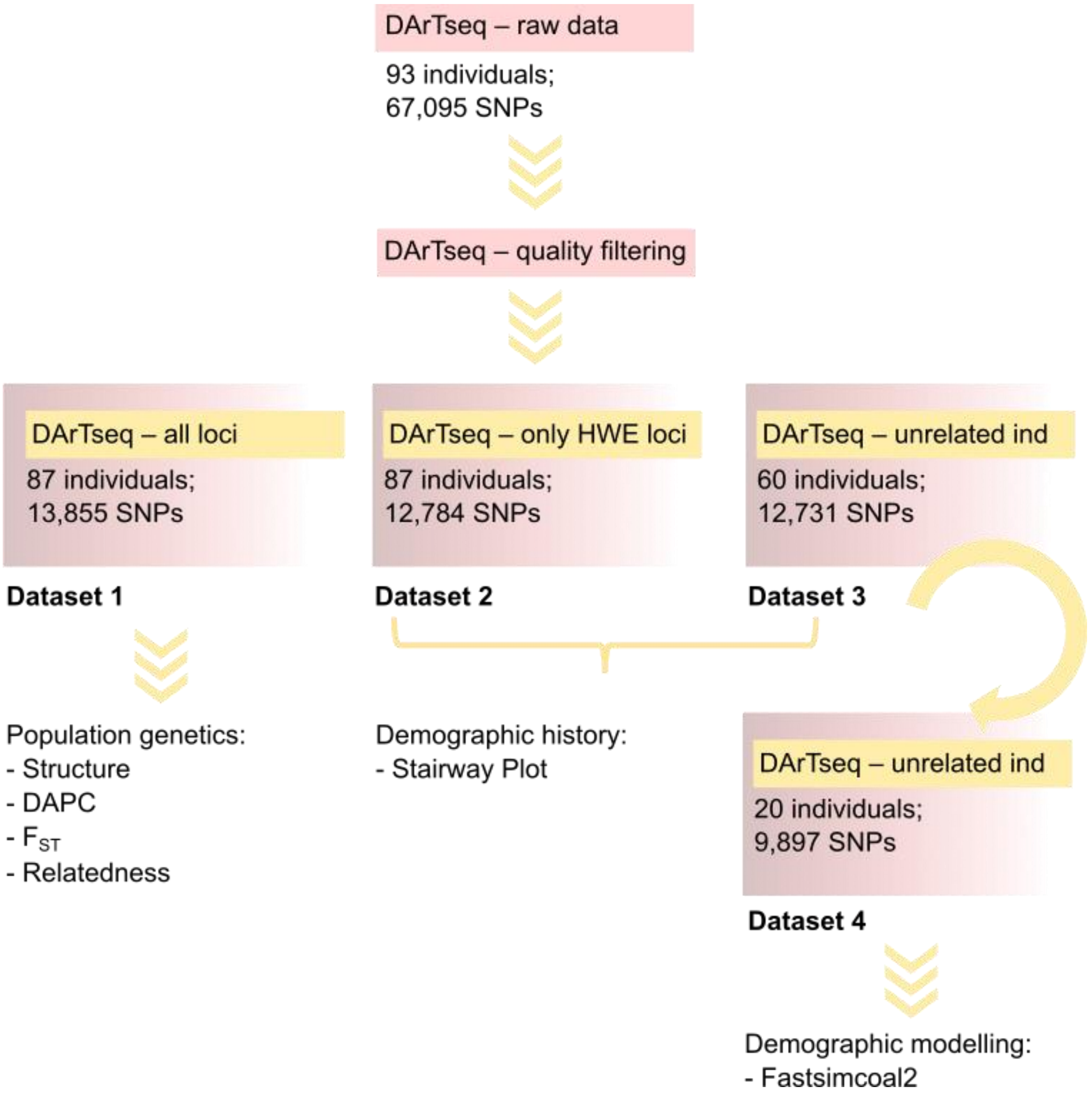

Fig. S8. Workflow of the data filtering process from the raw DArTseqTM read output (67,095 SNPs; 93 individuals) to the genomic datasets used for the downstream analyses (dataset 1 – 4).

Supplementary Tables

Table S1. Metadata file containing information about all individuals genotyped under this study and respective sampling details (n = 93 birds). All individuals that passed the quality filters (n = 87) were considered in the downstream analyses. The *Stairway Plot* analyses were repeated without the close relatives (i.e., parent-offspring and full-sibs), and with a subset of individuals to control for differences in sampling size among the two colonies. A total of 20 unrelated birds (i.e., 10 from each colony) were selected for demographic modelling with *fastsimcoal2*. For details about the DArTseq workflow see Fig. S8. RIR = Rivière des Remparts; RDC = Rond Des Chevrons; GB = light-grounded birds; CA = artificial colony; M = male; F = female; Ad = adult; Juv = juvenile; Lat = latitude; Long = longitude; Repro = year of reproductive season.

									DArTseq				
**Sample ID**	**Ring**	**Site**	**Sex**	**Age**	**Lat (°N)**	**Long (°E)**	**Repro**	**month**	**Genotyped**	**Structure & relatedness (dataset 1)**	**Stairway Plot (all) (dataset 2)**	**Stairway Plot (unrelated) (dataset 3)**	**fastsimcoal2 (dataset 4)**
PN100	FX25159	AC	F	Ad	-21.2	55.5	2018	Feb	×				
PN90	GE68541	AC	F	Ad	-21.2	55.5	2018	Jan	×				
PN1	FX17686	GB	F	Ad	-21.0	55.7	2008	Aug	×	×	×	×	
PN10	GE61291	GB	F	Juv	-21.3	55.3	2013	Feb	×	×	×	×	
PN11	GE61293	GB	M	Juv	-21.3	55.5	2013	Mar	×	×	×	×	
PN12	GE61294	GB	F	Juv	-21.3	55.5	2013	Mar	×	×	×	×	
PN13	GE68536	GB	F	Juv	-21.3	55.4	2014	Jan	×	×	×	×	
PN14	GE68538	GB	F	Juv	-20.9	55.5	2014	Jan	×	×	×	×	
PN15	GE53975	GB	F	Juv	-21.2	55.5	2015	Nov	×	×	×	×	
PN16	GE62845	GB	F	Ad	-21.0	55.5	2015	July	×	×	×	×	
PN17	GE54895	GB	F	Juv	-21.1	55.2	2015	Mar	×	×	×	×	
PN18	GE54896	GB	F	Juv	-21.2	55.3	2015	Mar	×	×	×		
PN19	GE54898	GB	F	Juv	-21.2	55.4	2016	Apr	×	×	×	×	
PN2	GE53118	GB	M	Juv	-21.3	55.5	2008	Jan	×				
PN38	NA	GB	F	Juv	-21.3	55.4	2016	Feb	×	×	×	×	
PN40	GE62567	GB	M	Juv	-21.3	55.5	2018	Jan	×	×	×		

PN48	GE62568	GB	M	Juv	-21.3	55.5	2017	Jan	×	×	×	×	
PN5	FX17649	GB	F	Juv	-21.2	55.5	2010	Feb	×				
PN6	FX17751	GB	F	Juv	-21.2	55.3	2010	Mar	×	×	×	×	
PN7	FX19835	GB	M	Juv	-21.2	55.5	2010	Apr	×	×	×	×	
PN70	GE65471	GB	F	Juv	-21.3	55.5	2018	Dec	×	×	×	×	
PN8	FX19792	GB	F	Juv	-21.3	55.6	2011	Mar	×	×	×	×	
PN9	GE61290	GB	M	Juv	-21.3	55.3	2013	Feb	×	×	×		
PN31	FX20997	RDC	F	Ad	-21.2	55.5	2016	Feb	×	×	×	×	
PN32	FX26517	RDC	M	Ad	-21.2	55.5	2016	Feb	×	×	×	×	
PN34	FX26518	RDC	M	Ad	-21.2	55.5	2016	Feb	×	×	×		
PN35	GE62057	RDC	M	Ad	-21.2	55.5	2016	Mar	×	×	×		
PN36	GE62069	RDC	M	Ad	-21.2	55.5	2016	Mar	×	×	×	×	×
PN37	FX20998	RDC	M	Ad	-21.2	55.5	2016	Mar	×	×	×		
PN44	GE62089	RDC	F	Ad	-21.2	55.5	2017	Oct	×	×	×	×	×
PN49	FX25121	RDC	M	Ad	-21.2	55.5	2017	Nov	×	×	×	×	
PN50	GE62058	RDC	M	Ad	-21.2	55.5	2017	Oct	×	×	×	×	×
PN51	GE62090	RDC	M	Ad	-21.2	55.5	2017	Oct	×	×	×	×	×
PN57	FX25120	RDC	M	Ad	-21.2	55.5	2017	Nov	×	×	×		
PN61	FX25146	RDC	F	Ad	-21.2	55.5	2017	Feb	×	×	×	×	
PN63	FX25147	RDC	F	Juv	-21.2	55.5	2017	Feb	×	×	×	×	×
PN64	FX25144	RDC	F	Juv	-21.2	55.5	2017	Feb	×	×	×	×	×
PN65	FX25143	RDC	M	Ad	-21.2	55.5	2018	Feb	×	×	×	×	
PN71	FX24648	RDC	F	Ad	-21.2	55.5	2018	Nov	×	×	×	×	
PN72	FX24649	RDC	M	Ad	-21.2	55.5	2018	Nov	×	×	×	×	
PN73	FX24650	RDC	F	Ad	-21.2	55.5	2018	Dec	×	×	×	×	×
PN76	FX24742	RDC	F	Ad	-21.2	55.5	2018	Dec	×	×	×	×	
PN77	FX24743	RDC	F	Ad	-21.2	55.5	2018	Dec	×	×	×	×	×
PN78	FX24744	RDC	M	Ad	-21.2	55.5	2018	Dec	×	×	×	×	
PN79	FX24745	RDC	M	Ad	-21.2	55.5	2018	Dec	×	×	×	×	×
PN84	FX25140	RDC	F	Ad	-21.2	55.5	2018	Dec	×				
PN86	FX25150	RDC	F	Ad	-21.2	55.5	2018	Dec	×	×	×	×	

PN87	FX25165	RDC	M	Ad	-21.2	55.5	2018	Dec	×	×	×		
PN88	FX25166	RDC	F	Ad	-21.2	55.5	2018	Sep	×	×	×		
PN89	FX25167	RDC	M	Ad	-21.2	55.5	2018	Dec	×	×	×		
PN93	FX24748	RDC	M	Ad	-21.2	55.5	2018	Jan	×	×	×	×	
PN94	FX24750	RDC	F	Ad	-21.2	55.5	2018	Jan	×	×	×	×	
PN95	FX24754	RDC	M	Ad	-21.2	55.5	2018	Jan	×	×	×		
PN96	FX24755	RDC	M	Ad	-21.2	55.5	2018	Feb	×	×	×	×	×
PN97	FX24756	RDC	M	Ad	-21.2	55.5	2018	Feb	×	×	×	×	
PN20	GE54899	RIR	M	Ad	-21.3	55.6	2016	Nov	×	×	×		
PN22	GE62053	RIR	F	Ad	-21.3	55.6	2016	Nov	×	×	×	×	
PN23	GE62054	RIR	M	Ad	-21.3	55.6	2016	Nov	×	×	×		
PN24	GE62065	RIR	F	Ad	-21.3	55.6	2016	Nov	×	×	×		
PN25	GE62066	RIR	F	Ad	-21.3	55.6	2016	Nov	×	×	×	×	
PN26	GE62068	RIR	M	Ad	-21.3	55.6	2016	Dec	×	×	×		
PN27	FX26513	RIR	M	Ad	-21.3	55.6	2016	Feb	×	×	×	×	
PN28	FX26514	RIR	M	Ad	-21.3	55.6	2016	Feb	×	×	×	×	
PN29	FX26515	RIR	M	Ad	-21.3	55.6	2016	Feb	×	×	×	×	×
PN30	FX26516	RIR	M	Ad	-21.3	55.6	2016	Feb	×	×	×	×	
PN39	GE62063	RIR	M	Ad	-21.3	55.6	2017	Aug	×	×	×		
PN41	GE62055	RIR	F	Ad	-21.3	55.6	2016	Nov	×	×	×	×	×
PN42	GE62067	RIR	F	Ad	-21.3	55.6	2016	Dec	×	×	×	×	
PN43	GE62084	RIR	M	Ad	-21.3	55.6	2017	Oct	×	×	×	×	×
PN45	GE62070	RIR	M	Ad	-21.3	55.6	2017	Oct	×	×	×		
PN46	GE62060	RIR	M	Ad	-21.3	55.6	2017	Oct	×	×	×		
PN47	GE62085	RIR	M	Ad	-21.3	55.6	2017	Oct	×	×	×	×	×
PN52	GE62061	RIR	F	Ad	-21.3	55.6	2017	Oct	×	×	×	×	×
PN53	GE62088	RIR	M	Ad	-21.3	55.6	2017	Oct	×	×	×	×	
PN54	GE62087	RIR	M	Ad	-21.3	55.6	2017	Oct	×	×	×		
PN55	FX25126	RIR	M	Ad	-21.3	55.6	2017	Nov	×	×	×		
PN58	GE62059	RIR	F	Ad	-21.3	55.6	2017	Oct	×	×	×		
PN59	FX25134	RIR	F	Ad	-21.3	55.6	2017	Oct	×	×	×	×	

PN60	FX25123	RIR	F	Ad	-21.3	55.6	2017	Nov	×	×	×	×	
PN66	FX25131	RIR	F	Ad	-21.3	55.6	2017	Feb	×	×	×	×	
PN67	FX25130	RIR	M	Juv	-21.3	55.6	2017	Feb	×	×	×		
PN68	FX25132	RIR	M	Ad	-21.3	55.6	2017	Feb	×	×	×		
PN74	FX24739	RIR	F	Ad	-21.3	55.6	2018	Oct	×	×	×		
PN75	FX24741	RIR	F	Ad	-21.3	55.6	2018	Oct	×	×	×	×	×
PN80	FX24747	RIR	M	Ad	-21.3	55.6	2018	Jan	×	×	×	×	×
PN81	FX25133	RIR	M	Ad	-21.3	55.6	2018	Sep	×	×	×	×	×
PN82	FX25138	RIR	M	Ad	-21.3	55.6	2018	Sep	×	×	×	×	×
PN83	FX25139	RIR	F	Ad	-21.3	55.6	2018	Nov	×				
PN85	FX25149	RIR	M	Ad	-21.3	55.6	2018	Aug	×	×	×		
PN91	GE54900	RIR	M	Ad	-21.3	55.6	2016	Nov	×	×	×		
PN92	FX24746	RIR	F	Juv	-21.3	55.6	2018	Jan	×	×	×	×	
PN98	FX24757	RIR	F	Ad	-21.3	55.6	2018	Feb	×	×	×		
PN99	FX24758	RIR	M	Ad	-21.3	55.6	2018	Feb	×	×	×	×	×
								**N total**	**93**	**87**	**87**	**60**	**20**

Table S2. Ranking of the seven demographic models tested with *fastsimcoal2* for *Pseudobulweria aterrima* based on the Akaike Information Criteria (AIC). Log_{10} Likelihoods (Log10(Lhood)) were computed based on the parameters that maximized the likelihood of each model in a total of 100 independent simulations per model, in log_{10} units. ΔLhood represents the difference between the observed and expected Likelihood for the best simulation. ΔAIC corresponds to the difference in AIC to the best demographic model. The models M3 and M7 exhibited the lowest Log_{10} Likelihood and AIC values.

	Model	Log_{10}(Lhood)	Δ Lhood	# parameters	AIC	ΔAIC	Rank
M1	Null model	-9212.639	150.6	3	42439.4	410.3	7°
M2	Bottlenecks without gene flow	-9195.036	133.0	7	42366.3	337.3	5°
M3	Bottlenecks with gene flow	-9121.815	59.8	7	42029.1	0.0	1°
M4	Asymmetric bottleneck	-9134.802	72.8	5	42084.9	55.8	3°
M5	Ancient bottleneck	-9204.598	142.6	5	42406.4	377.3	6°
M6	Two ancient bottlenecks	-9177.008	115.0	7	42283.3	254.2	4°
M7	Ancient & recent bottlenecks	-9121.429	59.4	9	42031.3	2.2	2°

Table S3. Demographic parameter estimates that maximized the likelihood for each demographic model after 100 independent simulations per model (M1 – M7). Population size parameters are given in haploid numbers and time estimates in number of generations. The parameters estimated by the two best ranked models (M3; *bottlenecks with gene flow* and M7; *ancient & recent bottlenecks*) are very congruent and suggest a similar demographic history for Mascarene petrel for the last two millennia. See Table S6 for parameters definition.

	**Model**	N_{RIR}	N_{RDC}	$N1_{RIR}$	$N1_{RDC}$	TB_{RIR}	TB_{RDC}	T_{SPLIT}	TB_{ANC1}	TB_{ANC2}	N_{ANC2}	N_{ANC3}
M1	Null model	265	494	_	_	_	_	24	_	_	_	_
M2	Bottlenecks without gene flow	29	194	3814	636	2	2	34	_	_	_	_
M3	Bottlenecks with gene flow	18	18	1710	142	2	1	92	_	_	_	_
M4	Asymmetric bottleneck	34	69	_	1699	3	_	130	_	_	_	_
M5	Ancient bottleneck	56	106	_	_	_	_	5	1457	_	523694	_
M6	Two ancient bottlenecks	194	542	_	_	_	_	18	426	2776	183	835626
M7	Ancient & recent bottlenecks	11	32	1662	124	1	4	78	1980	_	720816	_

Table S4. Confidence intervals (CI) for parameter estimates under the two best demographic models (M3, *bottlenecks with gene flow*; and M7; *ancient & recent bottleneck*) with *fastsimcoal2*. Maximum-likelihood (ML) estimates were obtained from the run with the highest composite likelihood (see Fig. 3). The 95% confidence intervals (CI) were generated from 40 bootstrap replicates estimated following [9]. Population size estimates are given in number of haploid copies, and time estimates in number of generations. In bold: ML estimates that lie within the 95% CI. RIR = *Rein de Dimitile* colony; RDC = Rond Des Chevrons; N_e = Effective population size; N_{RIR} = N_e of RIR at present time; N_{RDC} = N_e of RDC at present time; $N1_{RIR}$ = N_e of RIR after the fragmentation of the ancestral population into two colonies; $N1_{RDC}$ = N_e of RDC after the fragmentation of the ancestral population into two colonies; N_{ANC2} = N_e of the ancestral population before size changes; T_{split} = Time of the fragmentation of the ancestral population into RIR and RDC; Tb_{RIR} = Time when RIR underwent a reduction on population size and connectivity; Tb_{RDC} = Time when RDC underwent a reduction on population size and connectivity; Tb_{ANC1} = Time when the ancestral population underwent a bottleneck; 2_{Nm} = average number of haploid immigrants entering the population per generation, where 2_{Nm0} denotes recent gene flow and 2_{Nm1} ancient gene flow among the two colonies. 2_{Nm0} = 0.01; 2_{Nm1} = 0.5.

M3 ("bottlenecks with gene flow")			
		95% CI	
Parameter	ML estimate	lower	upper
NRIR	**18**	16	18
NRDC	18	21	27
N1RIR	**1710**	1521	1713
N1RDC	**142**	131	161
TBRIR	**2**	1.7	2.1
TBRDC	1	1.2	2.0
TSPLIT	**92**	84	98

M7 ("ancient & recent bottleneck")			
		95% CI	
Parameter	ML estimate	lower	upper
NRIR	11	18	24
NRDC	**32**	27	37
N1RIR	1662	1348	1557
N1RDC	124	132	154
TBRIR	1	2.0	2.9
TBRDC	4	1.7	3.1
TSPLIT	78	82	91
TBANC1	1980	3169	4257
NANC2	720816	401014	553188

Table S5. Quality filtering steps applied to the DArTseq dataset for loci and individuals. Analyses were performed using the *dartR* v 2.1.4 [10] *R* package. SNPs = Single Nucleotide Polymorphism.

Filter	Function	# Individuals	# SNPs
Start	–	93	67,095
Remove individuals from the artificial colony (CA)	–	91	66,795
Loci trimmed from the sequence tag along with the adaptor	gl.filter.overshoot	91	66,563
Loci on the same sequence (short-distance linkage)	gl.filter.secondaries	91	43,470
Individuals call rate ≥ 0.80	gl.filter.callrate.ind	91	43,470
Individual heterozygosity ≥ 0.13	gl.report.het.FDD	87	43,470
Monomorphic loci	gl.filter.monomorphs	87	43,204
Locus call rate ≥ 0.90	gl.filter.callrate.loc	87	29,722
Loci reproducibility ≥ 95%	gl.filter.reproducibility	87	28,114
Coverage ≤ 10 reads and ≥ 40 reads	gl.filter.rdepth	87	15,500
Minor Allele Frequency (MAF) ≤ 0.01	gl.filter.maf	87	14,088
Sex-linked loci (t.het = 0.25; t.hom = 0.25)	gl.filter.sexlinked	87	14,066
Outlier loci	gl.outflank; Pcadapt	87	13,855
Post population structure analyses: departure from HWE (α = 0.01)	gl.filter.hwe	87	12,784
Post population structure analyses: 1°-degree relatives	gl.filter.hwe	60	12,731

Table S6 List of demographic parameters used for each model with *fastsimcoal2* and respective search ranges. Population size parameters are given in haploid numbers and time estimates in number of generations. None of the parameters were bound. RIR = Rivière des Remparts; RDC = Rond Des Chevrons; GB = light-grounded birds; N_e = Effective population size.

Parameter	Interpretation	Models	Value Type	Distribution Type	Search Range	
					**Min.**	**Max.**
N_{RIR}	N_e of RIR at present time	1, 2, 3, 4, 5, 6, 7	Integer	Uniform	1	500
N_{RDC}	N_e of RDC at present time	1, 2, 3, 5, 6, 7	Integer	Uniform	1	500
N_{RDC}	N_e of RDC at present time	4	Integer	Uniform	1	2000
$N1_{RIR}$	N_e of RIR after ancestral population become fragmented	2, 3, 4, 7	Integer	Uniform	100	2.00E+03
$N1_{RDC}$	N_e of RDC after ancestral population become fragmented	2, 3, 7	Integer	Uniform	100	2.00E+03
N_{ANC2}	N_e of the ancestral population before size changes	1, 5	Integer	Uniform	100	1.00E+06
N_{ANC2}	N_e of the ancestral population before size changes	6	Integer	Uniform	100	1.00E+04
N_{ANC2}	N_e of the ancestral population before size changes	7	Integer	Uniform	1000	1.00E+06
N_{ANC3}	N_e of ancestral population before the most ancient bottleneck	6	Integer	Uniform	1.00E+04	1.00E+06
Tb_{RIR}	Time when RIR underwent a bottleneck	2, 3, 4, 7	Integer	Uniform	1	15
Tb_{RDC}	Time when RDC underwent a bottleneck	2, 3, 7	Integer	Uniform	1	15
T_{split}	Time when the ancestral population become fragmented in RIR and RDC	1	Integer	Uniform	1	8000
T_{split}	Time when the ancestral population become fragmented in RIR and RDC	2, 3, 4, 5, 6, 7	Integer	Uniform	15	40
Tb_{ANC1}	Time when the ancestral population underwent a bottleneck	5, 7	Integer	Uniform	40	8000
Tb_{ANC1}	Time when the ancestral population underwent a 1° bottleneck	6	Integer	Uniform	40	2500
Tb_{ANC2}	Time when the ancestral population underwent a 2° bottleneck	6	Integer	Uniform	2500	8000

Supplementary References

1. Coffman, A. J., Hsieh, P. H., Gravel, S. & Gutenkunst, R. N. Computationally Efficient Composite Likelihood Statistics for Demographic Inference. *Mol. Biol. Evol.* **33**, 591–593 (2015).
2. Jacquard, A. *The genetic structure of populations*. vol. 5. Springer Science & Business Media (2012).
3. Blouin, M. S. DNA-based methods for pedigree reconstruction and kinship analysis in natural populations. *Trends Ecol. Evol.* **18**, 503–511 (2003).
4. Bienvenu, F. & Legendre, S. A new approach to the generation time in matrix population models. *Am. Nat.* **185**, 834–843 (2015).
5. Grzegorcyk, E. Dynamique de la population d'un oiseau marin endémique et menacé, le pétrel de Barau. Université Paris Saclay (2019).
6. Jombart, T., Devillard, S. & Balloux, F. Discriminant analysis of principal components: a new method for the analysis of genetically structured populations. *BMC Genet.* **11**, 1–15 (2010).
7. Evanno, G., Regnaut, S. & Goudet, J. Detecting the number of clusters of individuals using the software STRUCTURE: A simulation study. *Mol. Ecol.* **14**, 2611–2620 (2005).
8. Devloo-Delva, F. *et al.* Accounting for kin sampling reveals genetic connectivity in Tasmanian and New Zealand school sharks, Galeorhinus galeus. *Ecol. Evol.* **9**, 4465–4472 (2019).
9. Meier, J. I. *et al.* Demographic modelling with whole-genome data reveals parallel origin of similar Pundamilia cichlid species after hybridization. *Mol. Ecol.* 123–141 (2017) doi:10.1111/mec.13838.
10. Mijangos, J. L., Gruber, B., Berry, O., Pacioni, C. & Georges, A. dartR v2: An accessible genetic analysis platform for conservation, ecology and agriculture. *Methods Ecol. Evol.* **13**, 2150–2158 (2022).
